# Spatially distributed computation in cortical circuits

**DOI:** 10.1126/sciadv.abl5865

**Published:** 2022-04-22

**Authors:** Sergei Gepshtein, Ambarish S. Pawar, Sunwoo Kwon, Sergey Savel’ev, Thomas D. Albright

**Affiliations:** 1 Systems Neurobiology Laboratories, Salk Institute for Biological Studies, La Jolla, CA, USA.; 2 Center for Spatial Perception and Concrete Experience, University of Southern California, Los Angeles, CA, USA.; 3 Herbert Wertheim School of Optometry & Vision Science, University of California Berkeley, Berkeley, CA, USA.; 4 Department of Physics, Loughborough University, Loughborough, UK.

## Abstract

The traditional view of neural computation in the cerebral cortex holds that sensory neurons are specialized, i.e., selective for certain dimensions of sensory stimuli. This view was challenged by evidence of contextual interactions between stimulus dimensions in which a neuron’s response to one dimension strongly depends on other dimensions. Here, we use methods of mathematical modeling, psychophysics, and electrophysiology to address shortcomings of the traditional view. Using a model of a generic cortical circuit, we begin with the simple demonstration that cortical responses are always distributed among neurons, forming characteristic waveforms, which we call neural waves. When stimulated by patterned stimuli, circuit responses arise by interference of neural waves. Results of this process depend on interaction between stimulus dimensions. Comparison of modeled responses with responses of biological vision makes it clear that the framework of neural wave interference provides a useful alternative to the standard concept of neural computation.

## Teaser

Investigating interference of neural waves helps to overcome limitations of the traditional view of cortical computation.

## INTRODUCTION

The traditional view of neural computation in the cerebral cortex holds that sensory neurons are each characterized by selectivity for certain dimensions of sensory stimuli, such as their orientation, direction of movement, and spatiotemporal frequencies. This view has been augmented by evidence of nonlinear “contextual” interactions between stimulus dimensions (*1*–*8*). A notable example of the latter is selectivity of neurons in middle temporal (MT) cortical area of alert monkeys (*9*), where neuronal preferences for stimulus spatial frequency (SF) were found to depend on stimulus luminance contrast, more so when luminance contrast was a slowly varying function of time. Here, we propose a mechanism that can account for this form of contextual influence on neuronal selectivity.

To study this mechanism, we developed a spatially distributed model of cortical computation in which an excitatory-inhibitory circuit (*10*, *11*) is used as a motif repeated across neural tissue and connected to form a chain. We show that responses of this model are always distributed among neurons, forming characteristic waveforms that we term neural waves. Waves elicited by different parts of the stimulus spread across the chain and interfere with one another. Patterns of interference created by this process have a number of characteristics that determine the circuit’s preference for stimulation. Notably, the circuit’s preference for stimulus SF predicted by this method is expected to depend on both luminance contrast and temporal properties of the stimulus, resulting in patterns of interaction between stimulus dimensions that are markedly similar to those found in MT neurons, as described above (*9*).

By investigating other properties of interference patterns, here, we make additional novel predictions about selectivity of cortical circuits and about contextual interaction between stimuli. We confirm these predictions using physiological and behavioral methods. We show in particular that neuronal preferences for stimulus temporal frequency (TF) depend on stimulus SF, which is a hitherto unknown phenomenon predicted by the model. We also show that the modulated activity that arises in the visual system outside of the region of direct stimulation does not depend on the SF of the visual stimulus, consistent with the model prediction about interference of neural waves. These findings suggest that the framework of neural wave interference offers a useful predictive account of neural computation in visual cortical circuits, which we offer for consideration as an alternative to the account of computation by neurons characterized by sustained specialization.

Our study contributes to the larger body of theoretical and empirical investigation of wave phenomena in neural systems, which have attracted considerable theoretical and empirical interest. Pertinent to our work, previous theoretical studies explored such phenomena as propagation of activity in spatially structured networks (*12*) and formation of patterned activity in neural fields, again by means of traveling waves (*13*, *14*). These investigations suggested that traveling waves can contribute to formation of stimulus selectivity in cortical mechanisms, such as directional selectivity (*15*), and that interaction between patterns of activity propagating across cortex can perform computations (*16*), although these predictions have not yet received empirical confirmation. Previous empirical studies concentrated primarily on how wave phenomena coordinate cortical operations: in terms of creating synchronous patterns of activity between neural circuits that underlie various behavioral states (*17*–*20*) and in terms of transfer of information between circuits (*21*–*25*), rather than on computational properties of wave phenomena.

Here, we combine theoretical and empirical methods to better understand computational properties of wave phenomena. By means of mathematical analysis, we demonstrate that neural waves create a distributed spatiotemporal pattern and that a study of interference of these waves allows one to predict perceptual and physiological properties of biological vision. We then show that this approach helps to understand visual phenomena that appear to be puzzling from the traditional perspective of neuronal specialization.

## RESULTS

Our results consist of two threads, theoretical and empirical. In the theoretical thread, we examine spatial interactions between neurons using a distributed model of a generic cortical circuit. In the empirical thread, we test several predictions of this model using sensory psychophysics in human subjects and single-neuron physiology in alert macaque monkeys.

### Model of distributed circuit

#### Approach

**Fig. 1.**
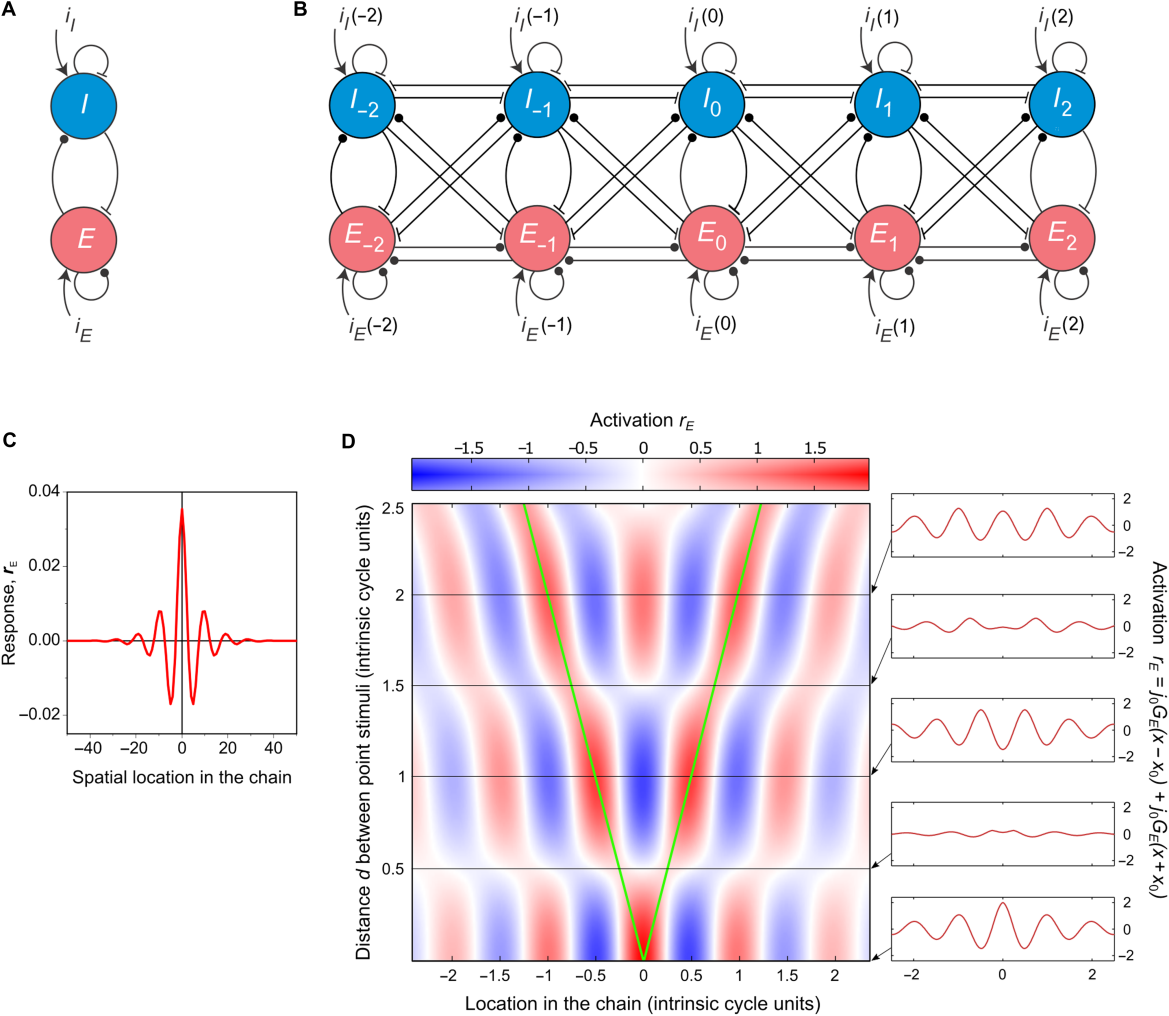
Distributed canonical circuit and its intrinsic tuning to SF. (**A**) The generic Wilson-Cowan circuit contains one excitatory unit (*E*) and one inhibitory unit (*I*), connected reciprocally, each with recurrent feedback. The lines ending with circles and T-junctions represent excitatory and inhibitory connections, respectively, between the cells. (**B**) The generic circuit is used as a motif in a distributed circuit. Indices *l* of the motifs (notated as *E_l_* and *I_l_*) indicate motif locations in the circuit. Currents *i_E_*(*l*) and *i_I_*(*l*) are the inputs into each motif generated by the stimulus. (**C**) Response of the distributed circuit to a small point stimulus that activates a single motif in the circuit. The resulting point activation propagates through the circuit and forms a steady-state pattern: a neural wave that has the form of spatially damped oscillations. Only the excitatory component of the neural wave is shown. (**D**) Interference of neural waves originating in two locations in the circuit, each activated by a point stimulus. The plot portrays a map of activation distributed across circuit locations (the abscissa) for multiple distances *d* between two point stimuli (the ordinate). The activated locations are represented by green lines: starting at the same location at the bottom (*d* = 0). The red and blue entries in the map represent positive and negative activation, explained in the color bar. The insets at the right are horizontal sections through the map at five interstimulus distances (expressed in units of the intrinsic period of the circuit). As in (C), only excitatory components of neural waves are shown.

We developed a spatially distributed model of cortical computation based on the excitatory-inhibitory circuit introduced by Wilson and Cowan (*10*, *11*). Their original circuit is illustrated in Fig. 1A. It consists of excitatory (*E*) and inhibitory (*I*) parts connected reciprocally and recurrently. Extensive studies of this circuit suggested that it can serve as a generic precursor for dynamic models of biological neural networks (*8*, *19*, *26*–*31*).

In our model, this circuit is used as a motif. The motifs are repeated across neural tissue and connected to form a chain: a spatially distributed form of the basic excitatory-inhibitory circuit. In this minimal model, we only consider a one-dimensional arrangement of motifs since it provides a simple preparation for investigating spatially distributed phenomena. We consider complete connectivity between neighboring motifs (Fig. 1B), in which the excitatory and inhibitory cells of each motif are each connected to both excitatory and inhibitory cells of neighboring motifs.

An emergent property of this spatially distributed circuit is an intrinsic preference for the distributed stimuli that serve as input to the system. We begin by illustrating the mechanism that underlies this intrinsic preference, in three steps. First, we use a localized “point” stimulus that activates a single motif and elicits a spatially distributed response: a “standing” neural wave. Second, we consider two spatially distinct point stimuli that activate two motifs, which are separated by distances that allow interference between the resulting neural waves. Last, we investigate circuit responses to spatially distributed stimuli and compare model predictions in linear and nonlinear regimes with results of psychophysical and physiological studies of biological vision. We find that the model of a fully connected circuit can successfully explain phenomena of lateral interaction between stimuli and stimulus selectivity of cortical circuits.

#### Neural wave interference

We begin by considering a very small (point) stimulus that initially activates a single motif in the chain. The point activation propagates through the chain by means of intermotif coupling. The stationary state of this process is a distributed spatial pattern of activation. We found the shape of this pattern by integrating the equations in which the circuit is represented as a continuous system (Eq. 2). The solution can be usefully described as a neural wave with two components: excitatory and inhibitory. Both components are distributed through the network, and both have the form of a damped wave with the same SF and the same rate of decay (damping). An example of the excitatory component is shown in Fig. 1C; it is a standing spatially damped wave (Eq. 3). The components interact, making them parts of a single neural wave.

A larger stimulus activates multiple parts of the network. The neural waves originating in different locations in the network form a complex distributed pattern. Assuming linearity (an assumption we relax in the last section of Results), the resulting pattern can be predicted by the principle of superposition, where the distributed activation is the sum of multiple waves activated at different points, forming a pattern of spatial interference. Activation is expected to be higher or lower depending on whether the interference of waves is constructive or destructive (illustrated below; Fig. 1D). We use the intuition of linear interference of neural waves to illustrate how the distributed circuit becomes intrinsically selective for stimulus SF.

Suppose the chain is activated by two spatially separated identical point stimuli, each generating a neural wave originating at a unique motif. By virtue of interference of these waves, circuit activity between the point stimuli is facilitated or suppressed at different locations. Figure 1D is a map of the excitatory component of the neural wave distributed over multiple locations in the chain (represented on the abscissa) for different distances *D* between the point stimuli (represented on the ordinate). Notice that the response pattern is periodic, but the period does not depend on the interstimulus distance. This period is determined by the weights of excitatory and inhibitory connections between cells; it is an intrinsic property of the circuit (henceforth “intrinsic period” or its inverse: “intrinsic frequency”).

The variety of resulting interference patterns can be seen by examining horizontal slices through the map, as shown at the right in Fig. 1D. When the two point stimuli overlap at the zeroth location (*D* = 0, bottom inset), the positive activation is highest at the location of these stimuli, forming a waveform of positive and negative activations at other locations. Increasing distance *D* (moving upward on the ordinate) leads to alternating states of network activity. When *D* is proportional to the intrinsic period of the chain, overall activation is heightened because the waves of activation from the two stimulated locations “arrive” in phase with one another, as in the third and fifth insets from the bottom where the stimuli are separated by 1 and 2 intrinsic periods, respectively. When distance *D* is proportional to half the intrinsic period, by contrast, the overall activation is reduced because the waves arrive in antiphase and cancel one another, as in the second and fourth insets from the bottom, where the stimuli are separated by 0.5 or 1.5 intrinsic periods, respectively.

#### Direct and lateral activation in the model circuit

**Fig. 2.**
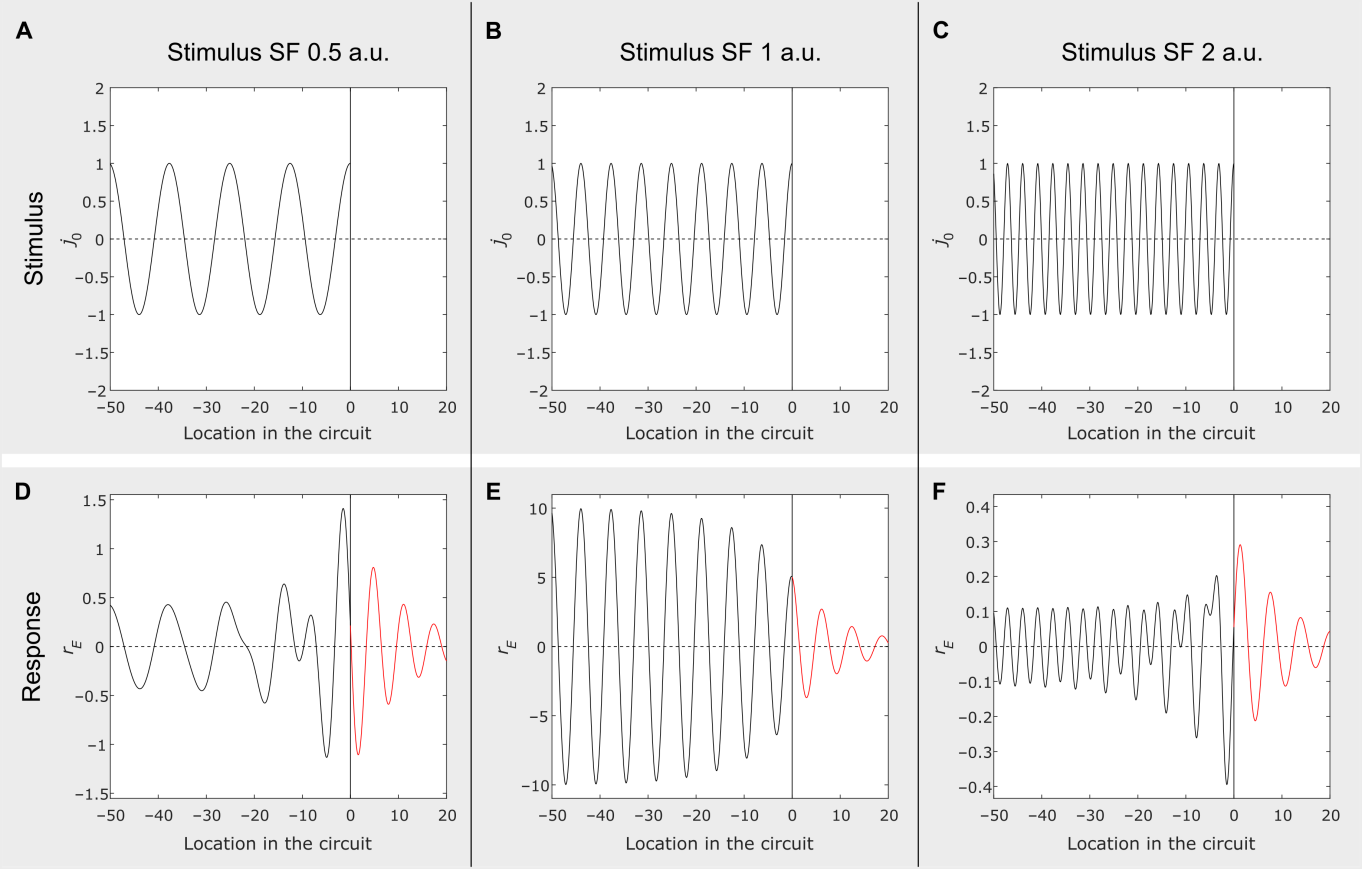
Response of the model circuit to luminance gratings. (**A** to **C**) Three grating stimuli at the spatial frequencies of 0.5, 1, and 2 units of the intrinsic SF of the circuit. Stimulation is applied only to the part of the circuit that corresponds to negative values of the abscissa, with no direct stimulation for its positive values. (**D** to **F**) Simulated responses of the model circuit to stimuli in the same column: for circuit locations stimulated directly (in black) and for circuit locations outside of direct stimulation (in red). The responses were derived using Eqs. 3 and 4. As in Fig. 1 (C and D), only excitatory components of neural waves are shown. a.u., arbitrary units.

Next, we investigated the response of the model to distributed spatial stimuli. In this case, the neural waves elicited on multiple cells in the chain interfere and form a distributed pattern, as they do for the simpler two point stimulation described above. Here, we considered a common situation in which the stimulus is spatially continuous over a segment of the chain, but it is bounded: stopped abruptly by a well-defined edge. In the top row of Fig. 2, we plot three examples of such a stimulus: a luminance grating at three SFs. The analytical solution for the model response to each stimulus can be found by convolving the stimulus with the model response to the point stimulus. The excitatory component of the solution is plotted in the bottom row of Fig. 2. This analysis revealed that response properties are qualitatively different in the region of the model circuit where the cells are activated directly (henceforth “zone 1”) and outside of that region (“zone 2”).

##### Zone 1

Well within zone 1 (shown in Fig. 2 in black), the SF of the response waveform is equal to the stimulus SF (SF_stim_). Near the boundary of zone 1, but still inside of it, we find a mixture of two frequencies: SF_stim_ and intrinsic SF of the system (abbreviated below as SF*). Away from the boundary, response amplitude depends on how close SF_stim_ is to SF*. The amplitude is maximal when SF_stim_ approaches SF* (spatial resonance).

##### Zone 2

Just outside of the region of direct stimulation, we find a “lateral activation” of the circuit (shown in red). Here, the response SF changes abruptly to SF*, but the amplitude of response gradually decreases as a function of distance away from the boundary of direct stimulation. The SF of the response in zone 2 is independent of the stimulus SF.

In the following sections, we evaluate the suitability of the model by empirically testing some of its key predictions. In the next section, we use psychophysical methods to test the model prediction that the SF of lateral activation in zone 2 does not depend on the stimulus SF. In subsequent sections, we examine predictions concerned with circuit’s stimulus selectivity in zone 1.

### Lateral activation in human vision

Numerous psychophysical studies have found that visual systems change their sensitivity outside of regions of direct stimulation (*32*–*35*). These results are often attributed to long-range “horizontal” connections between cortical neurons (*36*–*38*). With rare exception (*39*), lateral activation is probed using spatially distributed stimuli, such as “Gabor patches.” Responses to distributed probes arise by integration of multiple neural waves, reflecting the structure of the probe in addition to the neural waves generated by other stimuli (Eq. 4). To avoid this problem, we obtained psychophysical measurements of lateral activation using a small structureless probe.

**Fig. 3.**
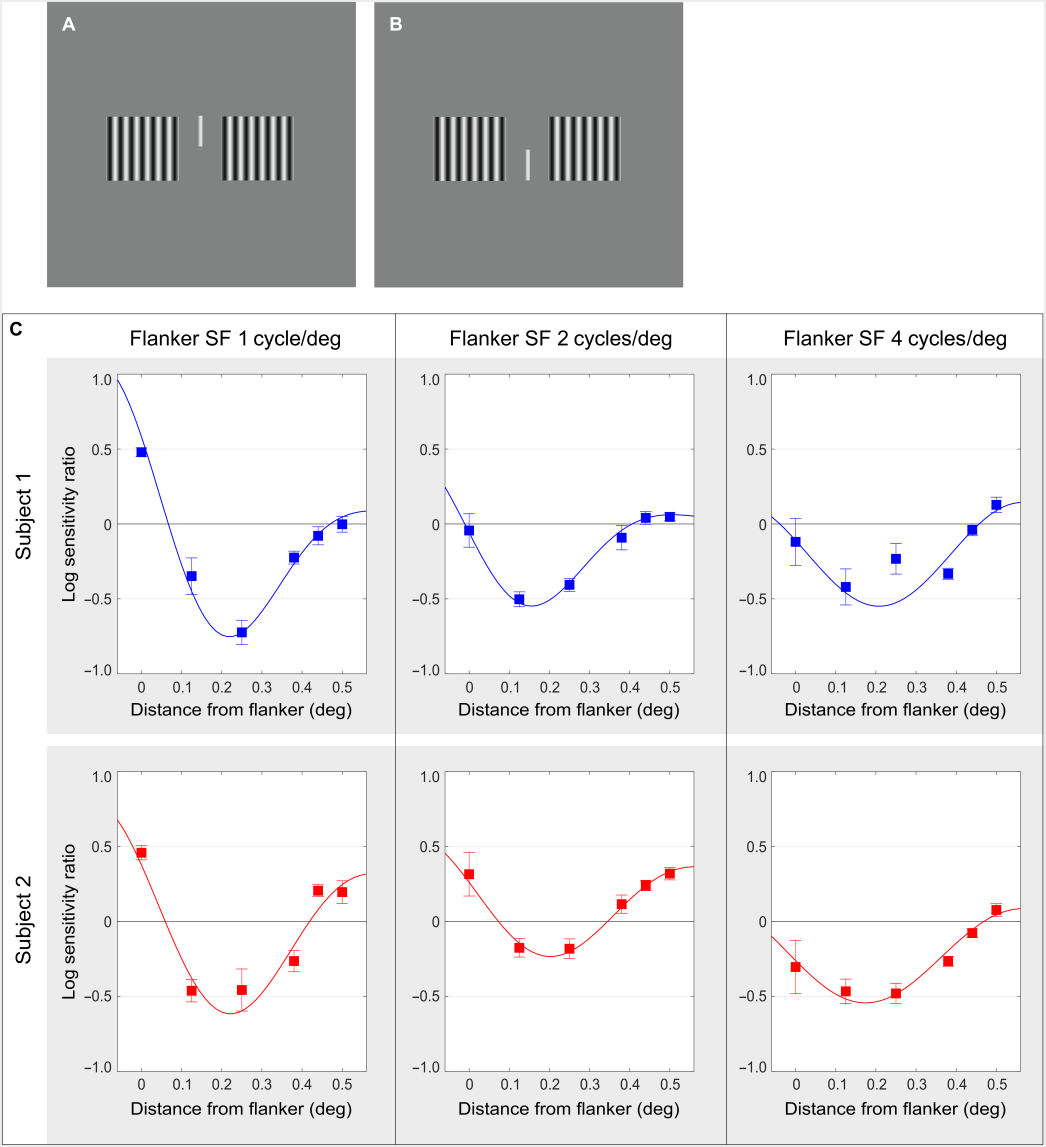
Measured contrast sensitivity in the region of lateral activation. (**A** and **B**) Stimulus used in the psychophysical study. The probe was a faint vertical line (shown here as a high-contrast line) presented between two square patches of luminance grating (flankers). The probe appeared either above (A) or below (B) the horizontal midline. The task was to report whether the probe was seen in the upper or lower position. (**C**) In each panel, contrast sensitivity to the probe in the region of lateral activation is plotted as a function of distance from the flankers. (The data for probes presented at the left and right of the screen center were collapsed into a single function of distance from flanker edge.) Each column of plots contains data for a different SF of the flankers, and each row of plots contains data for a different human subject. The curves are damped harmonic functions fitted to the data as described in Materials and Methods. The plots reveal a spatial waveform of lateral activation whose period does not systematically depend on the stimulus SF (see fig. S1). These results are consistent with the model prediction that lateral activation is periodic and that its SF is independent of the SF of direct stimulation.

The stimulus in our psychophysical study consisted of three parts: two inducing luminance gratings (“inducers”) and a faint vertical line (“probe”) positioned between the inducers (Fig. 3, A and B). The inducers were square patches of luminance gratings of fixed luminance contrast, whose abrupt edges ensured that the region of direct stimulation had a well-defined boundary (as in Fig. 2) and no direct subthreshold activation of the visual system was introduced outside of the visible part of the stimulus. We measured contrast sensitivity for the probe, which was placed between the inducers following the procedure described in Materials and Methods. The pattern of contrast sensitivity serves as an index of lateral activation.

Results of this experiment for two human subjects are displayed in Fig. 3 (C and D). The change in subjects’ contrast sensitivity to the probe is plotted as a function of distance from the nearest inducer edge for three values of inducer SF. The columns of plots contain data for different values of the inducer SF, and the rows contain data for different subjects.

The results displayed in Fig. 3 reveal a clear modulation of contrast sensitivity outside of the region of direct stimulation. These results are evidence that lateral activation of the human visual system yields a pronounced spatial waveform and that the period of this lateral spatial modulation does not systematically depend on the stimulus SF (see fig. S1), consistent with the prediction of our model. The results support the view that lateral activation of the visual system is constituted by neural waves.

### Selectivity of cortical circuits

In addition to eliciting lateral activity, neural waves in the model interact inside the region of direct stimulation, forming interference patterns that define the conditions of circuit resonance, at which the amplitude of response is maximal. The resonance SF (SFR) of the circuit has a value close to its intrinsic frequency (SF*) when wave decay over distance (spatial damping) in the system is low (Eq. 16). We studied conditions of circuit resonance in two ways. First, we took into account the fact that our stimuli are modulated in both space and time, and thus, they are characterized by both SF and TF. Using a linear approximation of the model, we investigated the conditions of spatial and temporal resonance that determine, respectively, spatial and temporal stimulus preferences of the circuit. Second, we investigated how conditions of resonance depend on stimulus contrast, which required that we study circuit behavior beyond linear approximation.

#### Spatiotemporal selectivity in the model circuit

##### Spatial selectivity

**Fig. 4.**
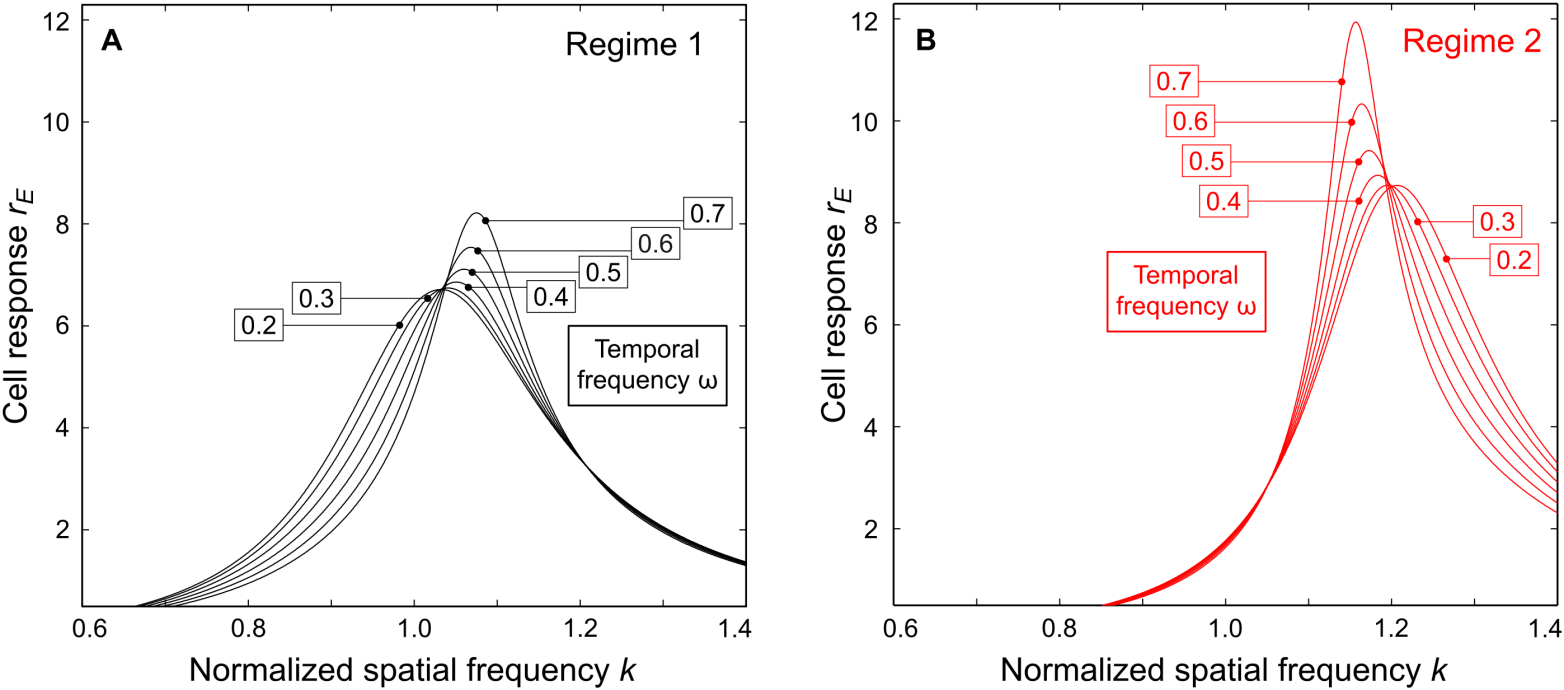
Maximum of model circuit response is predicted to depend on stimulus TF. (**A**) Circuit response functions are plotted for different TFs of the stimulus in regime 1. Function values are the amplitudes of neural waves computed using Eqs. 7 and 8. The values of stimulus TF are displayed in the boxes. The maximum of each response function is obtained at the SFR of the model circuit for the given stimulus TF. SFR increases with stimulus TF. (**B**) Response functions are plotted as in (A) for regime 2. Here, SFR decreases with stimulus TF.

**Fig. 5.**
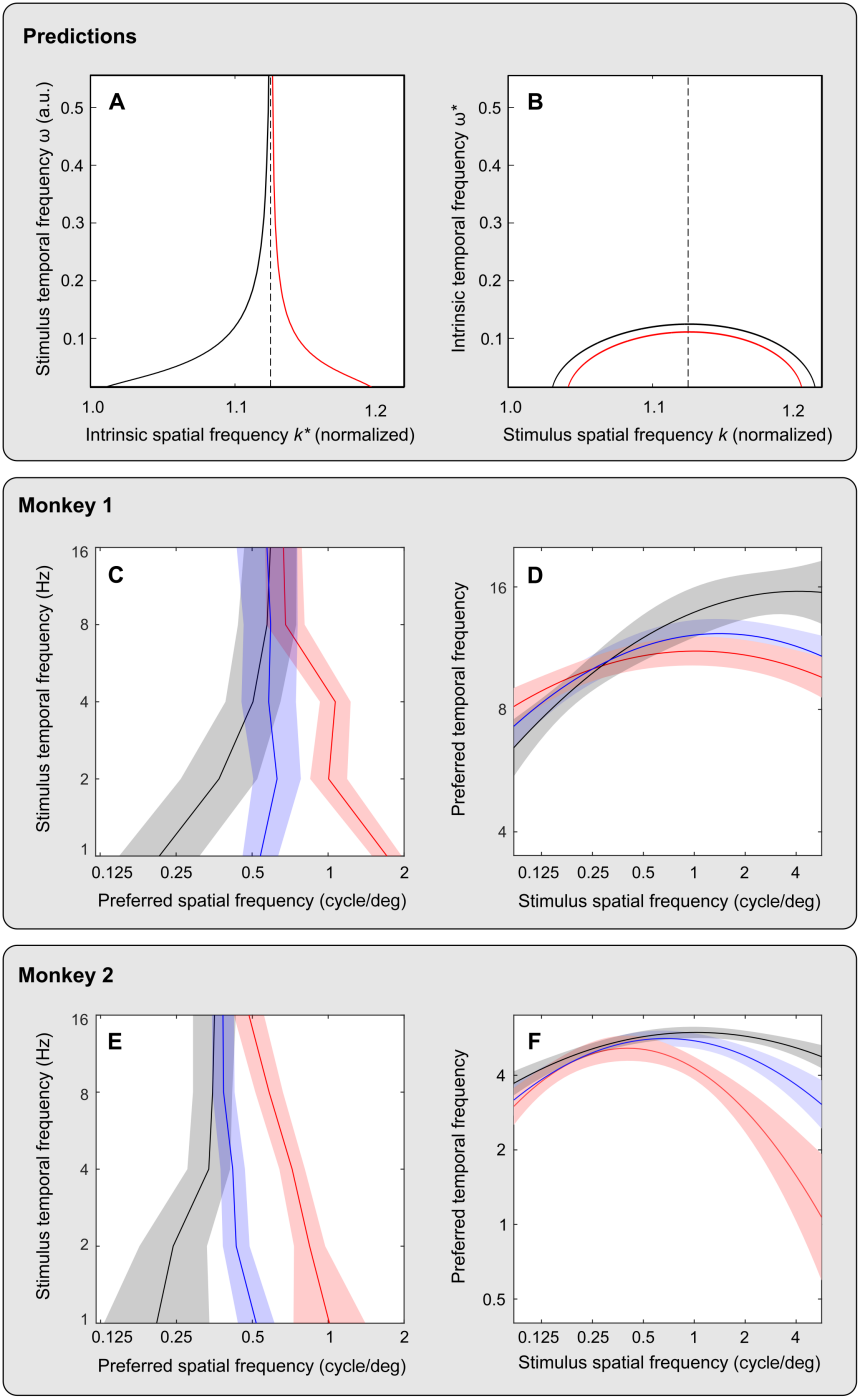
Spatial and temporal resonance in the model circuit and in macaque cortical neurons. (**A** and **B**) Predictions of SFRs for varying stimulus TF are displayed in (A) (summarizing maxima of response function in Fig. 4), and predictions of TFRs for varying stimulus SF are displayed in (B) (not shown in Fig. 4). The black and red curves represent two regimes of circuit activation. (**C** to **F**) Summaries of the preferred SF (C and E) and TF (D and F) measured in populations of neurons in area MT in two monkeys [monkey 1 in (C) and (D) and monkey 2 in (E) and (F)] for three stimulus contrasts. The lines and the shaded regions represent the means and errors of the estimates, respectively: for low contrasts in black and gray, for medium contrasts in blue and light blue, and for high contrasts in red and pink. (See Figs. 6 and 7 for details of neuronal data and stimulus parameters.)

Results of our analysis of SFR in the model (Eqs. 6 to 9) are summarized in Figs. 4 and 5A. We found that increasing stimulus TF leads to changes in spatial resonance (SFR). The form of this relationship depends on the weights of connections in the circuit, forming two qualitatively different patterns. In one pattern (which we call regime 1), SFR increases with TF; in the other pattern (regime 2), SFR decreases with TF.

Regime 1 is illustrated in Fig. 4A, where model responses are plotted for different values of stimulus TF, represented by separate curves. The peak of each response function is found at the SFR for the indicated stimulus TF. Response peaks shift rightward, toward higher SF values, as TF grows, represented in Fig. 5A by the black curve at the left. Regime 2 is illustrated in Fig. 4B. Here, response peaks shift leftward (toward lower SF values) as TF grows, represented in Fig. 5A by the red curve at the right.

The rise of SFR in regime 1 is bounded by a vertical asymptote (a separatrix) represented in Fig. 5A by a dashed vertical line. The decline of SFR in regime 2 is bounded by the same vertical asymptote. In summary, as TF increases, SFR approaches the asymptote from the left side in regime 1 and from the right side in regime 2. As weights change, system behavior changes continuously from one regime to another, passing through conditions in which SFR does not depend on TF.

This remarkable result also suggests a different explanation, in terms of interaction of excitatory and inhibitory components of the neural wave rather than in terms of weights between cells (Eq. 1 versus Eqs. 6 to 9). The formalism of interaction of wave components (which describes neural function in terms of distributed patterns of activity) helps to simplify the account of system preferences as compared to the formalism of weights between cells (which describes essentially local phenomena, such as interaction between individual cells). The approach of wave interaction allows one to reduce the number of variables used to describe the system: from the number of participating cells to the number of participating wave components (which is two components in a purely spatial formulation or four components in the spatiotemporal formulation, respectively; Eqs. 13 and 6). From the perspective of interaction of wave components, different regimes of circuit behavior (and change of circuit behavior from one regime to another) can arise because coefficients of interaction between neural wave components depend on stimulus TF (Eq. 6). (The formalism of relationship between strength of connections between cells, on the one hand, and coefficients of interaction between wave components, on the other hand, is described by Eqs. 8 to 10 and eqs. S12 to S15.)

##### Temporal selectivity

Next, we show how temporal resonance of the model [its resonance TF (TFR)] depends on stimulus SF. In this case, we vary stimulus TF for fixed values of stimulus SF to find the TF of maximal response (TFR). We perform this analysis of TFR for the same parameters of the model circuit at which we have obtained the solutions for SFR in the preceding section (Eq. 9). The predicted pattern of TFR (Fig. 5B) is radically different from the pattern of SFR in terms of the shape of interaction of spatial and temporal stimulus frequencies (Eq. 10). The prediction is that TFR should remain a nonmonotonic function of stimulus SF.

#### Spatiotemporal selectivity in biological cortical circuits

We tested predictions of our network model with regard to SF and TF selectivity in biological cortical circuits. The predictions summarized in Fig. 5 (A and B) were tested by applying new analyses to data that were obtained in a previously reported (*9*) physiological study of stimulus preferences in the cortical area MT of two awake macaque monkeys. A preview of the physiological results appears in Fig. 5 (C to F). The juxtaposition of theoretical and physiological results in Fig. 5 reveals a notable similarity of changes in system preference predicted by the model and measured in the monkey.

**Fig. 6.**
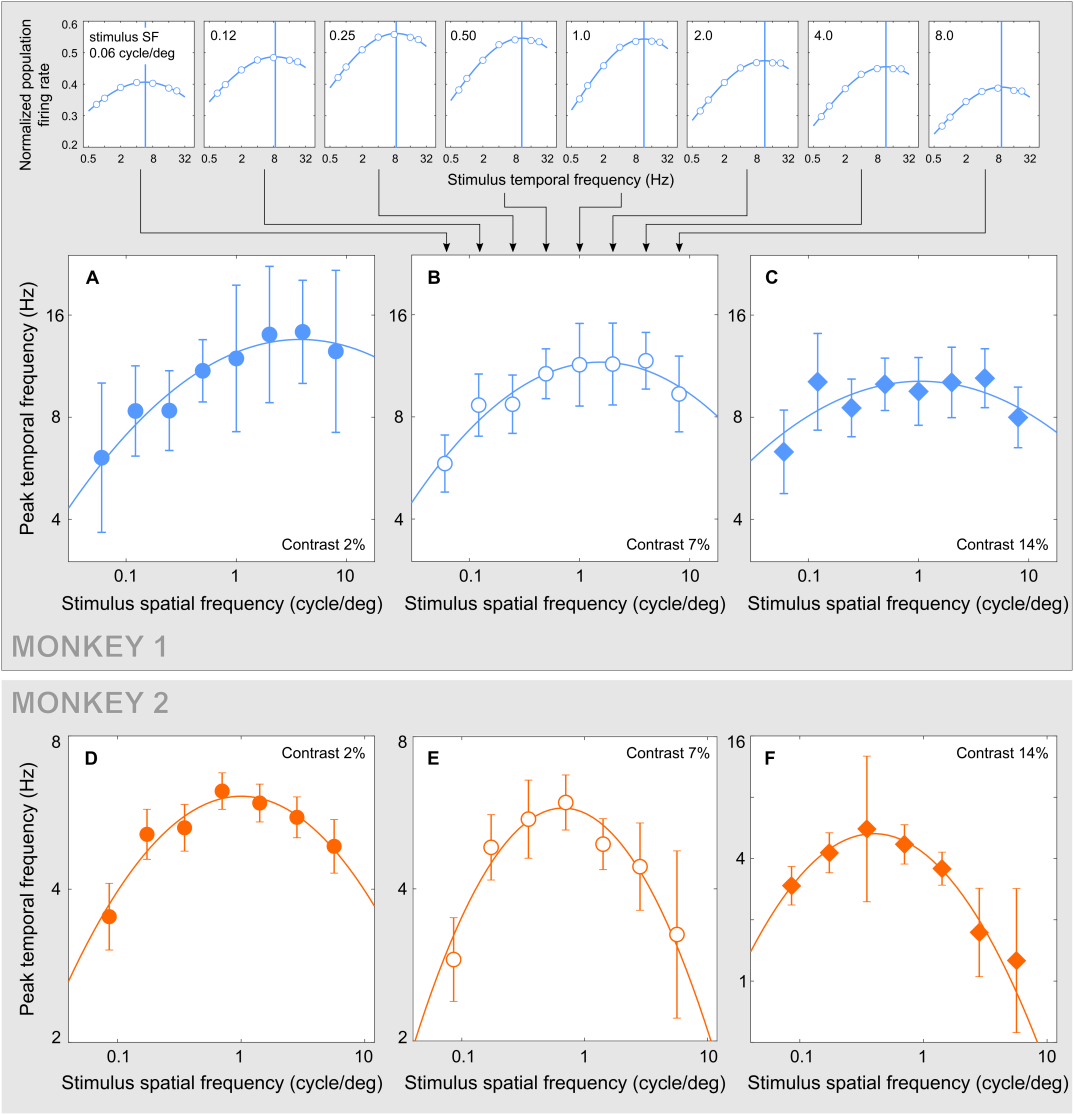
Preferred TF of MT neurons. The top row of plots contains response functions measured in populations of MT neurons in monkey 1 for eight stimulus SFs (marked at the top left of each panel), all at the stimulus contrast of 7%. The blue vertical line in each panel marks the peak TF assembled into a single function from eight stimulus SFs in (B). (**A** to **C**) Summaries of peak TF estimated at three stimulus contrasts (marked at the bottom right of each panel) for monkey 1. The error bars are SDs of peak TF estimated by resampling (Materials and Methods). (**D** to **F**) Results of the same analysis for monkey 2, in which peak TF could be estimated for in seven (rather than eight) conditions of stimulus SF. The shapes of these functions were as predicted by our model for contrasts 7 and 14% in monkey 1 (*P* = 0.008 and *P* = 0.004, respectively; Materials and Methods) and for all contrasts in monkey 2 (*P* ≪ 0.01 for contrasts 2 and 7% and *P* = 0.002 for contrast 14%). Note that the ordinate range in (F) is enlarged to accommodate the wider range of peak TF in this contrast condition. Numbers of neurons used to perform the measurements are reported in fig. S2.

**Fig. 7.**
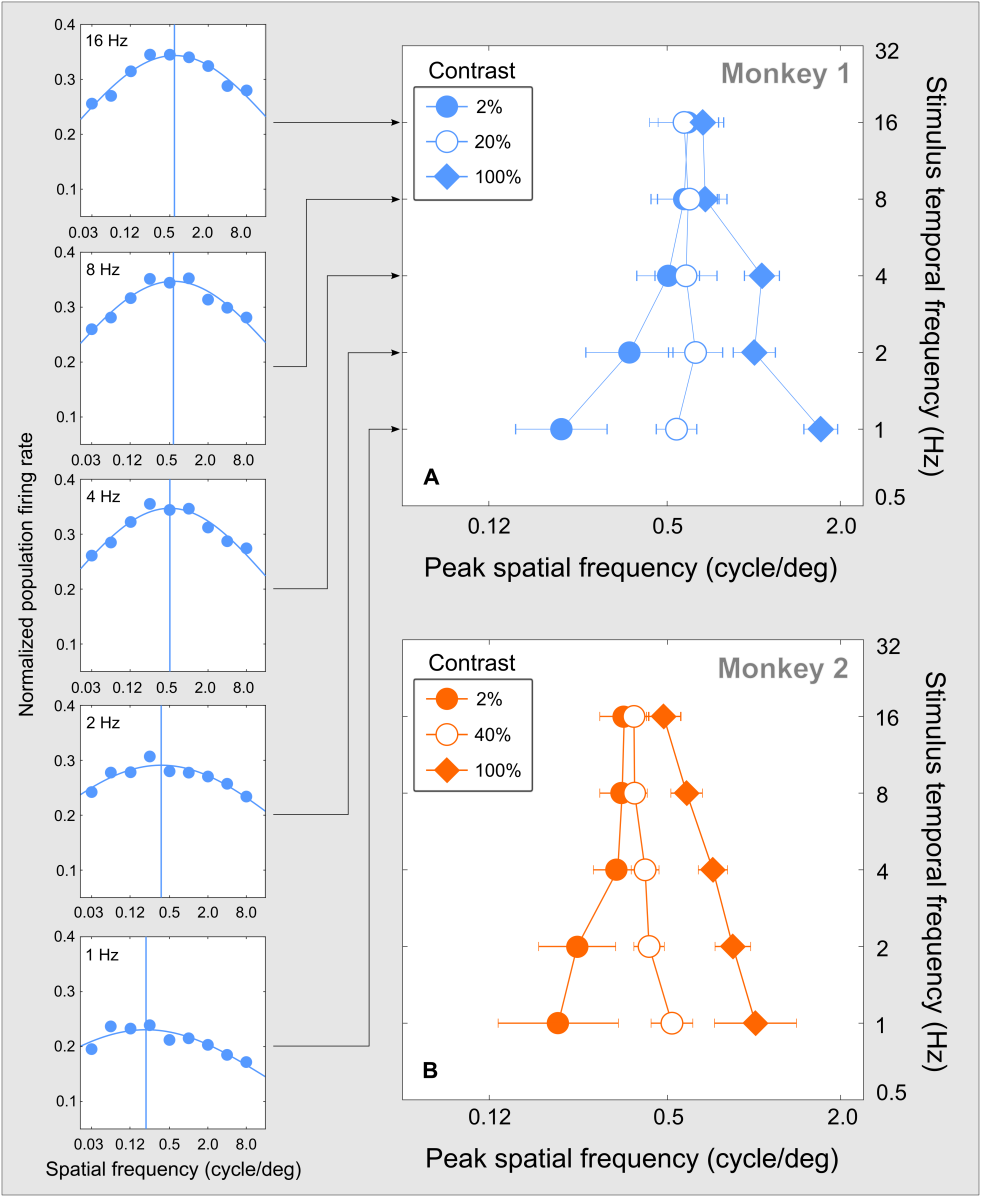
Preferred SF of MT neurons. (**A**) The left column of plots contains response functions measured in MT neurons of monkey 1 at the same stimulus contrast (2%) for five stimulus TFs (labeled at the top left of each panel). The blue vertical line in each panel marks an estimate of peak SF. The five estimates are assembled into a single function at the left (filled circles) in the larger graph. This graph contains two other functions of peak SF estimated in the same manner for stimulus contrasts of 20 and 100% in the same monkey. (**B**) Results of the same analysis for monkey 2. The results displayed for two monkeys were obtained in the same stimulus conditions, with the only difference that medium contrasts were 20% for monkey 1 and 40% for monkey 2. (Medium contrasts were chosen so that their plots occupied intermediate positions between the low- and high-contrast plots.) The error bars are SDs of peak SF estimated by resampling (Materials and Methods). Between stimulus contrasts of 2 and 100%, peak SF increased significantly in both monkeys: for TFs below 8 Hz in monkey 1 (*P* < 0.01; Materials and Methods) and for all TFs in monkey 2 (*P* < 0.03). Within contrast, peak SF increased with TF at the contrast of 2% (*P* < 0.01 in monkey 1 and *P* = 0.05 in monkey 2) and decreased with TF at the contrast of 100% (*P* < 0.01 in monkey 1 and *P* = 0.02 in monkey 2). Numbers of neurons used to perform the measurements are reported in fig. S3.

The stimuli used in the physiological study were drifting luminance gratings in which we varied SF, TF, and luminance contrast (henceforth “contrast”). Following the procedure described in Materials and Methods, we measured the firing rates of neurons for several values of stimulus SF at fixed stimulus TF and contrast, allowing us to estimate the neuron’s preference for stimulus SF. We then repeated this procedure for different fixed values of TF. By this means, we characterized each neuron’s spatial and temporal stimulus preferences for several values of stimulus contrast. We obtained these data from 74 neurons in monkey 1 and 66 neurons in monkey 2. In Figs. 6 and 7, we present results of an analysis of these data aggregated across all recorded neurons, separately for each monkey.

##### Temporal selectivity

Effects of stimulus SF on temporal selectivity of cortical neurons are illustrated in Fig. 6. Each plot in the top row of Fig. 6 represents the average neuronal firing rate (ordinate) for monkey 1 as a function of stimulus TF (abscissa), for a single value of stimulus SF (indicated at the top left in every plot) and a single stimulus contrast of 7%. By fitting the data across stimulus TF, we estimated the preferred TF for each stimulus SF, represented in each plot by a vertical line. A summary of these estimates for the stimulus contrast of 7% appears in Fig. 6B, revealing how the preferred TF of the population of cortical neurons varies with stimulus SF. Results of the same analyses for two other stimulus contrasts in monkey 1 are displayed in Fig. 6 (A and C), and results for the same three stimulus contrasts in monkey 2 are displayed in Fig. 6 (D to F). In both monkeys, the preferred TF follows a nonmonotonic function of stimulus SF. The shape of this function does not change with stimulus contrasts (confirmed for most conditions as detailed in the caption of Fig. 6), following the pattern predicted by our model (Fig. 5B).

##### Spatial selectivity

Effects of stimulus TF on spatial selectivity of cortical neurons are summarized in Fig. 7. Each plot in the left column of panels represents average neuronal firing rate (ordinate) for monkey 1 as a function of stimulus SF (abscissa), for a single value of stimulus TF (indicated at the top left in every plot) and a single stimulus contrast of 2%. By fitting the data across stimulus SF, we estimated the preferred SF for each stimulus TF, represented in each plot by a vertical line. A summary of these estimates for the stimulus contrast of 2% appears in Fig. 7A, together with estimates of preferred SF for two other stimulus contrasts. Results of the same analyses of data for monkey 2 are displayed in Fig. 7B.

The plots in Fig. 7 (A and B) indicate that increasing stimulus contrast led to a significant increase in the peak SF in both monkeys: for TFs below 8 Hz in monkey 1 and for all TFs in monkey 2. In addition, in both monkeys, peak SF increased with TF at low stimulus contrast and decreased with TF at high stimulus contrast. This pattern of change of preferred SF of cortical neurons is similar to model predictions (Fig. 5A) in which the shape of the SFR function changes radically: from an increasing function of TF in one regime to a decreasing function of TF in the other regime, while these functions converge with increasing TF.

In our modeling framework, these changes of cortical selectivity represent different instances of the same general pattern of dependency of SFR on stimulus TF. Beyond model predictions, the physiological results reveal that the separation of two regimes of interaction between peak SF and stimulus TF can be caused by stimulus contrast.

#### Stimulus contrast and cortical selectivity

**Fig. 8.**
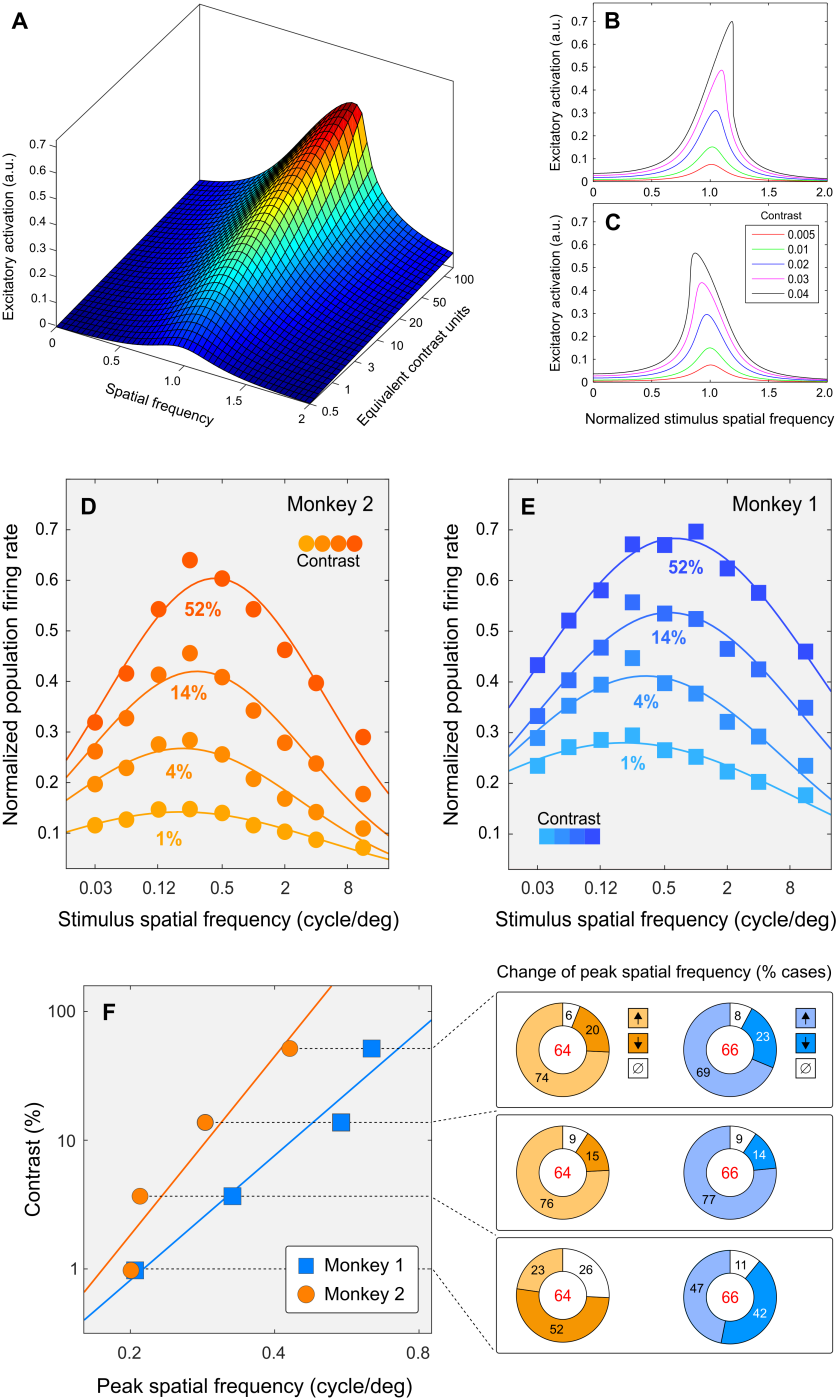
Response of the model circuit and cortical neurons to increasing stimulus contrast. (**A**) Theoretical response surface of the circuit obtained by iterating Eq. 15. (**B** and **C**) The curves are slices through the surface in (A). Each curve represents the amplitude of the excitatory component of neural wave in the model circuit at one stimulus contrast, plotted as a function of stimulus SF. Stimulus SF is normalized to the SFR of the circuit measured at the lowest tested contrast (the red curve). Increasing stimulus contrast can increase SFR, as in (A) and (B), or decrease it, as in (C) (see the “Linear resonance analysis” and “Nonlinear resonance analysis” sections in Materials and Methods, in particular Eq. 17). (**D** and **E**) Spatial response functions measured in populations of MT neurons are plotted separately for four stimulus contrasts (separated by color) for monkey 1 in (E) and monkey 2 in (D). The data are collapsed across TF. (**F**) Peak SFs of neural populations are plotted for four stimulus contrasts, summarizing results in (E) (monkey 1) and (D) (monkey 2), using colors matched by monkey. In both monkeys, peak SF increases with contrast in agreement with model predictions. The donut charts at the right display percentages of cases in which peak SF significantly (*P* < 0.05) increased (↑), decreased (↓), or did not change (∅) for each step of stimulus contrast. (Black numerals represent percentages and red numerals represent numbers of neurons used in the analysis.) Significance was established using the Wilcoxon rank-sum test.

Our physiological finding that the regime of system preference for SF depends on stimulus contrast suggests that our linear analysis of the circuit was too narrow. We therefore studied how system nonlinearity affects conditions of circuit resonance (Eqs. 11 to 17). This analysis made it clear that resonance frequency of the circuit depends on the interaction of excitatory and inhibitory components of neural waves. At low contrast, in the linear regime, the interaction of wave components depends on weights of cell connections and on stimulus SF and TF. At higher contrasts, however, the interaction becomes nonlinear, and it additionally depends on the amplitudes of the neural wave themselves (Eq. 15). SFR is predicted to decrease with contrast (Fig. 8C) if the cell weights of a particular neuron happen to cause its SFR to be high (and fall to the right of the asymptotic line in Fig. 5A). Conversely, SFR is predicted to increase with contrast (Fig. 8B) if its SFR at low contrast is low (and it falls to the left of the asymptotic line in Fig. 5A).

To illustrate this neural wave interaction, we plot in Fig. 8A the amplitude of the excitatory wave component as a function of both stimulus SF and contrast. Vertical slices through the surface, orthogonal to the contrast axis, correspond to individual SF response functions for different levels of stimulus contrast. Five of these slices are shown in Fig. 8B. In each case, responses follow a smooth unimodal function of SF. The maximum of each function identifies the SFR for the corresponding contrast. At low contrasts, where circuit behavior is approximately linear, SFR does not change with contrast (red and green curves). Increasing contrast leads to a uniformly higher activation of the circuit and, notably, to a change of SFR: its gradual increase (Fig. 8, A and B) or decrease (Fig. 8C).

Neuronal preferences for stimulus SF in area MT were shown to change with contrast (*9*) (also see Fig. 7 above). In an additional analysis, we derived neuronal population response functions using the aggregate estimates of firing rates, as in Figs. 6 and 7. These functions are plotted separately for four values of stimulus contrast in Fig. 8 (D and E). In both monkeys, the mean preferred SF increased with contrast, as shown at the left of Fig. 8F: Peak SF increased 3.11-fold in monkey 1 and 2.14-fold in monkey 2. This increase of peak SF in populations of neurons was accompanied by changes in individual-cell preferences summarized by six donut charts at the right of Fig. 8F. For example, the chart at the top right indicates that increasing stimulus contrasts from 14 to 52% in monkey 1 led to significantly increasing peak SF in 69% of cases and significantly decreasing peak SF in 23% of cases and caused no significant change in 8% of cases. The fraction of neurons in which peak SF increased with contrast itself increased with contrast, as evidenced by comparing the top and bottom panels at the right of Fig. 8F. In summary, we found that peak SF changed with contrast in a large majority of cortical neurons, consistent with the prediction of our model that SFR can increase or decrease with stimulus contrast. In the model, SFR increases or decreases with stimulus contrast because the interaction between excitatory and inhibitory components of the neural waves depends on stimulus contrast.

#### Generalizations of the model

##### Neural waves in two spatial dimensions

**Fig. 9.**
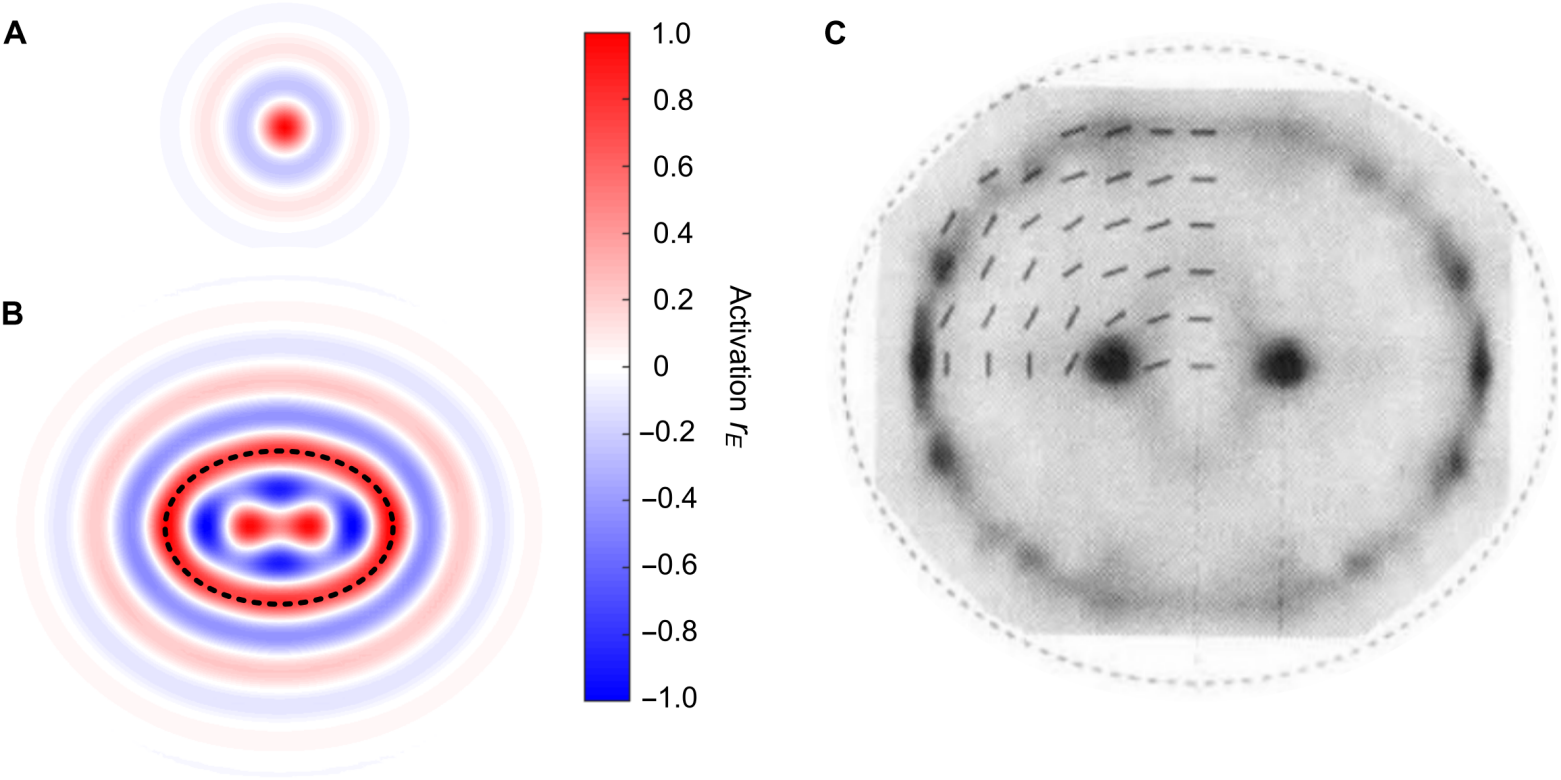
Interference of neural waves in two spatial dimensions. (**A**) Response of the two-dimensional excitatory-inhibitory circuit to a point stimulus (see Fig. 1C). Colors represent circuit activation *r_E_* explained in the color bar. The same color bar applies to (A) and (B). (**B**) Response of the model circuit to an elliptical ring stimulus (represented by the dashed black contour). Note two regions of positive activation near the focal points of the stimulus ring, predicting that contrast threshold should reduce in these regions as compared to other regions inside the ring. (**C**) Results of a psychophysical study in which a high-contrast elliptical ring was used as the stimulus (*26*). Gray levels represent contrast thresholds measured at multiple locations inside the ring. Dark colors represent reduction of threshold caused by the stimulus. Note that the threshold was reduced near the focal points of the ellipse, represented by two dark regions, in agreement with model predictions in (B). [Figure S4 is an illustration of how the shapes of these regions depend on neural threshold: the amount of neural activity required for stimulus detection. The higher the neural threshold, the more these two regions are separated, increasingly resembling the central part of (C)].

**Fig. 10.**
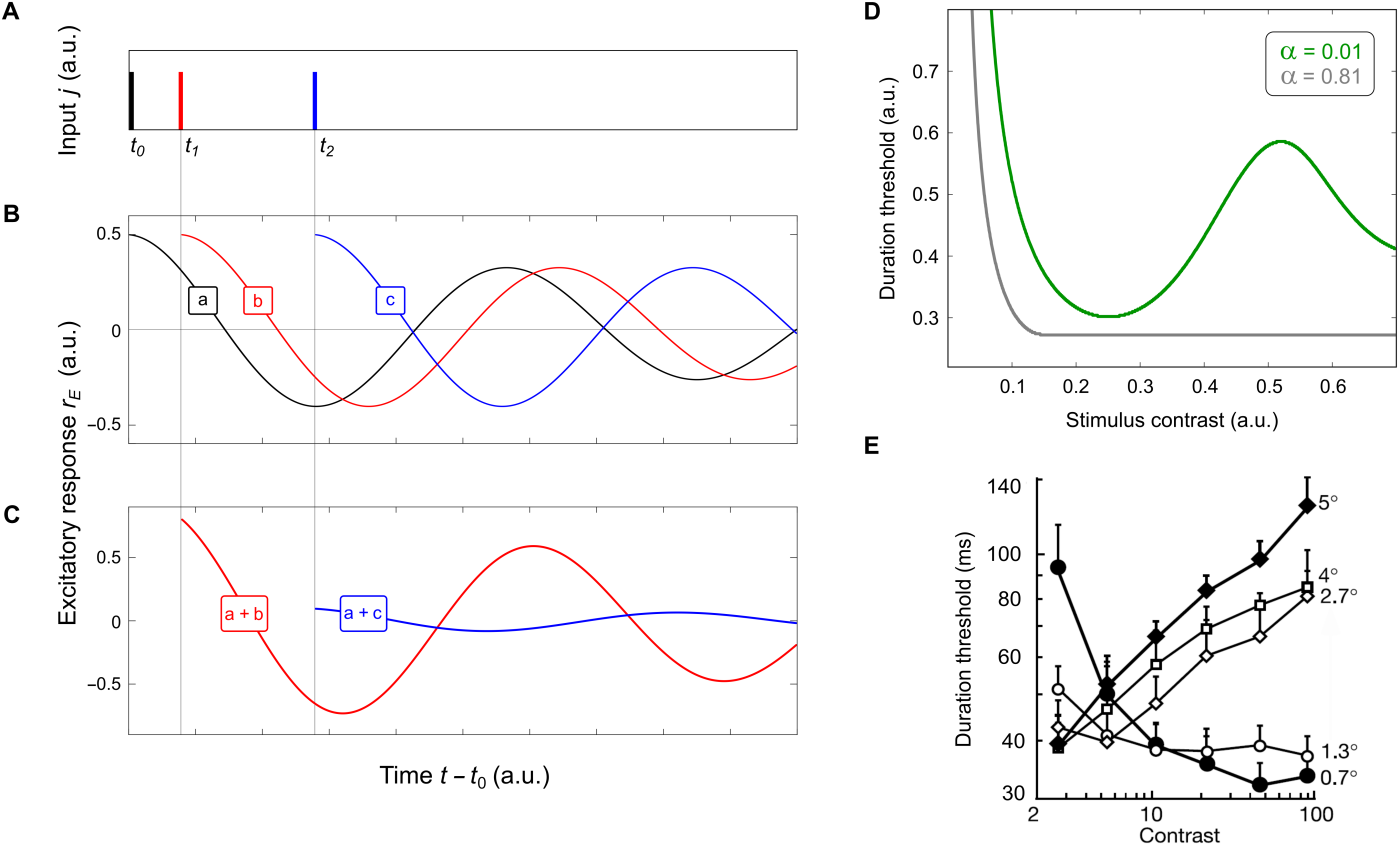
Interference of temporal oscillations and duration threshold. (**A** to **C**) Temporal oscillations “a,” “b,” and “c” (B) are elicited by impulses of brief stimulation at instants *t*_0_, *t*_1_, and *t*_2_ (A). The oscillations interfere with one another across time, forming patterns of activity of which two examples are shown in (C), obtained by superpositions “a + b” and “a + c.” These interference patterns add up to higher or lower activity, making the system more or less sensitive, respectively, to stimulation, during stimulation or after it. (**D**) By modeling temporal interference of oscillations in a single-node model (eq. S12), we found that stimulus visibility should depend on stimulus duration and luminance contrast. Effects of these parameters are predicted to interact, leading to counterintuitive results. Thus, when the circuit is dominated by excitation (model parameter α is high), stimulus duration at which the stimulus is just visible (duration threshold) is predicted to monotonically decline with stimulus contrast (gray curve). When the circuit is dominated by inhibition (α is low), duration threshold is predicted to form a nonmonotonic function of contrast (green curve). (**E**) Psychophysical studies (*47*–*48*, *52*) found that human duration threshold can increase or decrease with stimulus contrast depending on stimulus size (indicated at the right). Since stimulus size is known to determine the amount of inhibition in cortical circuits (*35*–*36*), our model can explain this reversal of relationship between stimulus contrast and duration threshold (*47*): from threshold decreasing with contrast for small stimuli [gray curve in (D)] to threshold increasing with contrast for large stimuli (green curve).

The model of wave interaction in neural circuits is readily generalizable to two spatial dimensions, suited to study cortical selectivity for stimulus orientation (*40*, *41*), shape (*33*, *35*, *42*, *43*), and stereoscopic depth (*44*, *45*). It is equally suited to study temporal interactions between successive stimuli (*34*, *46*, *47*) and the interaction between spatial and temporal dynamics of cortical activity (*48*, *49*), in cortical area MT and other cortical areas. Two examples of generalization of our model, spatial and temporal, are illustrated in Figs. 9 and 10.

In Fig. 9, we illustrate interference of neural waves in two spatial dimensions. We obtained these results by means of numerical simulation of activity in a two-dimensional lattice of nodes (eqs. S3 to S6), where each node was the same excitatory-inhibitory circuit as in our one-dimensional model (Fig. 1A). Node connectivity in this model has no asymmetry and anisotropy, and it does not vary across spatial location (eq. S5); these restrictions simplify our analysis here, yet they can be lifted in future studies. Neural waves originating in different locations in the network form a complex pattern of spatial interference, as in the one-dimensional model, except now this pattern is distributed in two spatial dimensions, even for a point stimulus (Fig. 9A). We used a high-contrast stimulus that had the shape of an elliptical ring, represented in Fig. 9B by a dashed black line. Interference of neural waves generated by this stimulus produced two salient regions of excitation near the two foci of the elliptical ring, clearly visible in the two-dimensional map of activation in Fig. 9B. Contrast threshold in these locations is predicted to be lower than in neighboring locations, in agreement with psychophysical findings (Fig. 9C) (*33*, *50*). The shape of the region of reduced threshold measured behaviorally (behavioral threshold) depends on the amount of neural activity required for stimulus detection (neural threshold), illustrated in fig. S4.

##### Nonstationary oscillations

In a different illustration, we studied interference of nonstationary neural oscillations across time (Fig. 10). Similar to how neuronal waves interfere across network locations, temporal oscillations generated at different instants in the network interfere across time. Activity produced at instant *t*_0_ will form a damped neural oscillation (e.g., “a” in Fig. 10B) across time *t* − *t*_0_ > 0. Activity produced at the same location at later instants *t*_1_ and *t_2_* will produce additional oscillations (“b” and “c” in Fig. 10B). Such subsequent oscillations superpose with one another, forming patterns of interference whose temporal profiles depend on whether individual oscillations generated at different instances are in phase (constructive interference) or antiphase (destructive interference) with one another (Fig. 10C).

One outcome of temporal interference of neural oscillations in our model is illustrated in Fig. 10D. We studied how duration and luminance contrast of a visual stimulus is predicted to affect its visibility across time. Intuitively, one expects that increasing stimulus duration and luminance contrast should make the stimulus more visible. Our study of interference of neural oscillations has suggested that, paradoxically, increasing stimulus duration can, in some cases, decrease visibility.

By numerically integrating responses of a single node of our model (Fig. 1A) over time, we investigated duration threshold: the stimulus duration at which the stimulus should become just visible (eqs. S7 to S10). In a system dominated by excitation, increasing contrast led to reduction of duration threshold, helping to make the stimulus more visible (i.e., requiring lower contrast to reach threshold). This result, represented in Fig. 10D by the gray curve, agrees with one’s intuition. However, in a system dominated by inhibition, represented in the figure by the green curve, increasing contrast leads to nonmonotonic changes of duration threshold, which fall or rise with contrast under different values of contrast. These properties of temporal interference can help to explain results of studies of duration threshold in human subjects (*46*, *47*, *51*), which revealed a similarly counterintuitive behavior. Duration threshold was found to rise or fall with stimulus contrast depending on stimulus size (Fig. 10E), which is known to control the amount of inhibition in cortical circuits (*52*, *53*).

In summary, two illustrations presented in Figs. 9 and 10 indicate that the model of neural wave interference has the potential to explain visual phenomena well beyond those reported in the empirical part of this study.

## DISCUSSION

We studied spatially distributed cortical computations by means of mathematical modeling, human psychophysics, and physiological recordings from isolated cortical neurons in nonhuman primates. We proposed a distributed model of cortical circuits that can account for some puzzling properties of the primate visual system and also predict properties not heretofore examined. Here, we discuss the possibility that interference of neural waves provides a general framework for understanding cortical computation.

We observed at the outset that point stimulation of the model circuit elicits a distributed periodic response: a neural wave with excitatory and inhibitory components that interact with one another. The natural spatiotemporal frequency of the neural wave is an intrinsic property of the circuit. When stimulation is distributed (i.e., applied to multiple points in the circuit), as is generally the case in biological vision, the neural waves evoked at different locations interfere with one another. This pattern of interference determines the stimuli to which the system resonates, thus defining the frequency selectivity of the model.

These results imply that the modeled excitatory-inhibitory circuits can function as spatiotemporal frequency filters, in agreement with the established notion that neural cells early in the visual system are tuned to SF and TF (*54*–*57*, *7*). In this latter view, early stages of the visual process are described as a bank of filters (*58*–*60*) selective for stimulus frequency, where parameters of individual filters (or parameters of the larger system) are readily estimated in psychophysical (*32*, *34*, *39*) and physiological (*56*, *57*) studies. In our framework, selectivity for frequency is only one property of a cortical mechanism characterized by a large repertoire of other noteworthy properties. In particular, we derived filtering properties of cortical networks in the form of the impulse response of equations describing these networks. We expect that a different analysis of the same equations will reveal how these networks can operate as a medium for propagating traveling waves, enabling the coordination of cortical processes at different spatial and temporal scales investigated in empirical studies of cortical waves (*22*, *24*, *25*, *61*, *62*).

In addition to establishing sensory selectivity in the region of direct stimulation, we have shown that neural waves propagate outside of the region of direct stimulation, causing lateral activation of the system. These two behaviors, concerned with system selectivity and its lateral activation, have been previously studied in the framework of separate mechanisms of classical and nonclassical receptive fields (*63*–*66*, *52*, *67*, *53*, *68*). Here, we proposed that these behaviors can arise from a single mechanism.

To assess the validity of this model, we empirically tested its specific predictions regarding stimulus selectivity and lateral activation in the primate visual system using physiological and psychophysical methods. Using physiological methods, we found that the changes of stimulus preference in biological cortical circuits depend on input stimulus parameters in a manner similar to such dependences in the model. In addition to our previous discovery that SF selectivity depends on stimulus contrast and TF (*9*), we report here that selectivity for stimulus TF depends on stimulus SF, which is a hitherto unknown phenomenon predicted by the model. Using psychophysical methods, we found that contrast sensitivity outside of the region of direct stimulation forms a modulated pattern whose SF is independent of stimulus SF. This finding supports the notion that lateral modulation of contrast sensitivity reflects an intrinsic neural wave.

These results suggest that our model of the distributed circuit based on a generic motif of excitatory-inhibitory interaction offers a useful account of biological vision, and that the language of interaction of neural waves affords explanation of phenomena of cortical selectivity that have resisted explanation in terms of specialized cortical neurons.

Our model of cortical connectivity is generic in the sense that we have only considered interactions of basic circuit components connected to form a distributed system. The model could apply equally to cortical areas other than MT as well as to information exchange between cortical areas. Attacking the question of whether stimulus interactions reported here are found in other cortical areas is an important direction for future empirical studies. Toward addressing this question, in the “Propagation of activity in a layered network” section in the Supplementary Materials, we consider a variant of our distributed network, in which the one-dimensional chain shown in Fig. 1B is replicated to form a series of parallel layers. The layers are connected so that activity can propagate from one layer to another, representing the sequence of information processing stages in biological visual systems. Our analysis of this system has demonstrated that neural waves can propagate between the layers while preserving the shape of the stimulus applied to the input layer, activated by the optical stimulus (eq. S11 and fig. S5). This result implies that responses of cortical networks at different stages in visual processing can be studied using the same spatial form of activation as the optical stimulus, as we did here.

Single-cell studies of stimulus selectivity in visual cortical areas revealed that primary visual cortex (area V1) is the largest source of input to area MT (*69*). Most of these projections from V1 to MT are selective for stimulus SF and TF just as are the cells in area MT. This evidence of similar stimulus preferences in areas V1 and MT is consistent with properties of the model of multilayered network, in which a broad band of subsequent layers is characterized by preference for stimulus frequency (fig. S5).

We have so far modeled only a limited version of neural wave interference. Beyond the single-chain model tested here, and the two-dimensional array model whose predictions we have not tested, our approach suggests a number of immediate generalizations. These generalizations include anisotropic properties of the network (allowing more complex forms of stimulus selectivity than those explored above) and interconnected neural arrays organized hierarchically (allowing additional computational benefits and for modeling the layered structure of the cortical tissue).

In addition to the improved understanding of biological computation, concepts of neural wave interference show much promise for developing new methods of “analog computation” (*70*), including artificial intelligence (AI) systems. Previous approaches to AI largely relied on artificial neural networks that learn by changing the strengths of connections between neurons. In contrast to this mainstream paradigm, spiking neural networks and oscillator-based computing (*71*) use the system’s rich repertoire of evolving dynamical states (*72*) to perform computations that offer significant energy savings (since spiking affords remarkably low expenditure of energy). Another unconventional computational paradigm of reservoir computing (*73*) requires that input information is prefiltered using a passive filter (which is not modified by learning) before signals enter a relatively small artificial neural network where learning takes place. The latter approach meets the demand for using small datasets for system training and retraining in place of the previous data-hungry AI algorithms where the entire larger system is retrained to accommodate a slightly modified task.

The approach of neural wave interference investigated here provides a framework for combining elements of oscillator-based computing and reservoir computing: deploying waves and oscillations for selectively highlighting (filtering) certain stimulus information, and offering varying dynamical states (such as synchronous oscillation of system parts or having the system approach or depart from dynamical attractors) for information processing and learning by means other than change of the strengths of connections between neurons. In this context, our work can be seen as a contribution to a new field of research that combines emerging concepts in neuroscience and AI (*74*, *75*) by hybridizing principally different learning paradigms: implemented by changing strengths of connections between parts of the network and by varying dynamical states of the system.

## MATERIALS AND METHODS

### Physiological methods

#### Animals

Two adult male rhesus monkeys (*Macaca mulatta*) of ages 11 and 12 were used in this study. Experimental protocols were approved by the Salk Institute Animal Care and Use Committee and conform to U.S. Department of Agriculture regulations and to the National Institutes of Health guidelines for the humane care and use of laboratory animals. Procedures for surgery and wound maintenance have been described in detail elsewhere (*9*, *81*).

#### Apparatus

All visual stimuli were generated using Matlab (MathWorks, Natick) software using a high-resolution graphics display controller (Quadro Pro Graphics card, 1024 × 768 pixels, 8 bits/pixel) operating in a Pentium class computer. Stimuli were displayed on a 21-inch monitor (75 Hz, noninterlaced, 1024 × 768 pixels; model GDM-2000TC; Sony, Tokyo, Japan). The output of the video monitor was measured with a PR650 photometer (Photo Research, Chatsworth, CA), and the voltage/luminance relationship was independently linearized for each of the three guns in the cathode ray tube.

#### Behavioral procedure

Monkeys were seated in a standard primate chair (Crist Instruments, Germantown, MD) with the head post rigidly supported by the chair frame. The task was to fixate a small (0.2° diameter) fixation target in the presence of moving visual stimuli for the duration of each trial (500 to 2000 ms). The target was presented on a video display at a viewing distance of 57 cm in a dark room (<0.5 cd/m^2^). The mean background luminance of the monitor was 15 cd/m^2^. Eye position was sampled at 120 Hz using an infrared video-based system (ISCAN, Burlington, MA). The eye position data were monitored and recorded with the CORTEX program (Laboratory of Neuropsychology, National Institute of Mental Health, Bethesda, MD), which was also used to implement the behavioral paradigm and to control stimulus presentation. After eye position was maintained within a 2° window centered on the fixation target throughout the trial, animals were given a small (0.15 ml) juice reward.

#### Electrophysiological procedure

A craniotomy was performed to allow electrode passage into area MT. Activity of single units was recorded in area MT using tungsten microelectrodes (3 to 5 megohms; Frederick Haer Company, Bowdoinham, ME), which were driven into cortex using a hydraulic micropositioner (model 650; David Kopf Instruments, Tujunga, CA). Neurophysiological signals were filtered, sorted, and stored using the Plexon (Dallas, TX) system. Visual responses were recorded from 139 directionally selective MT neurons in two awake fixating macaque monkeys (74 and 65 neurons in monkeys 1 and 2, respectively). We measured firing rates to stimuli at five to seven different levels of luminance contrast (0.05 to 100%) at the preferred spatiotemporal frequencies: five SFs and one to five TFs. The different stimulus conditions and contrasts were interleaved in random order across trials.

#### Data resampling

For each neuron, the firing rates estimated in separate trials within each condition of stimulus frequency and contrast were resampled with replacement. The number of samples was 10 (which is the number of trials used in the experiments). Response functions were fitted to the resampled data using nonparametric polynomial regression, repeated for 500 iterations of resampling to estimate errors of peak SF within each condition. The errors were used to measure differences between peaks across stimulus contrasts. A similar procedure was used to estimate errors of peak TF for each condition.

#### *Data analysis for* Fig. 6

Each point in the top plots of Fig. 6 was obtained by averaging firing rates within a sliding kernel defined on three stimulus dimensions: contrast, SF, and TF. For example, in the top left plot, the kernel contained four values of stimulus SF (geometric mean of 0.06 cycle/deg, indicated in the plot), three values of stimulus contrast (yielding the geometric mean of 7%), and one value of stimulus TF. The kernel was then advanced by one TF step while encompassing the same range of values of stimulus SF and contrast. Preferred values of stimulus TF were then estimated (marked by vertical lines in each top panel) and assembled into a temporal preference function (Fig. 6B). Three such preference functions are displayed in Fig. 6 (A to C) for stimulus contrasts of 2, 7, and 14% using monkey 1 data. The same analysis was performed using monkey 2 data (Fig. 6, D to F) as explained in the caption of Fig. 6.

#### *Data analysis for* Fig. 7

Similar to Fig. 6, a sliding three-dimensional kernel was used to average firing rates. For example, at the first point in the top left panel, the firing rate was measured for the kernel containing three values of stimulus TF (geometric mean of 16 Hz, indicated in the plot), two values of stimulus contrast (geometric mean of 2%), and three values of stimulus SF (geometric mean of 0.03 cycle/deg). The kernel was then advanced by one SF step while encompassing the same range of values of stimulus TF and contrast. Preferred values of stimulus SF were then estimated (marked by vertical lines in each panel at the left) and assembled into a spatial preference function. These preference functions are plotted in Fig. 7A for stimulus contrasts of 2, 20, and 100%, using monkey 1 data. The same analysis was performed using monkey 2 data for stimulus contrasts of 2, 40, and 100% (Fig. 7B) as explained in the caption of Fig. 7.

### Psychophysical methods

#### Subjects

Two adult subjects with normal or corrected-to-normal vision took part in the study. The subjects were given sufficient practice with the experimental task before the experiment.

#### Apparatus

The experiments were carried out in a dark room (<0.5 cd/m^2^). The stimuli were presented on a 21-inch monitor (Sony Color Graphic Monitor GDM-500PS) under the control of a personal computer with a commercially available circuit that provided high gray-scale resolution of 14 bits (*79*). Visual stimuli were generated by custom software and presented using the commercial software package Matlab (MathWorks, Natick). The monitor was gamma calibrated and had a resolution of 1600 × 1200 pixels (horizontal × vertical), with the vertical frame rate of 160 Hz. Subjects viewed stimuli binocularly from a distance of 57 cm using a chin-and-head rest.

#### Stimulus

The stimulus consisted of two square patches of vertical stationary luminance grating (“flankers”) and a vertical line (“probe”) positioned between the flankers. The mean luminance of the screen and of the flankers was 24.65 cd/m^2^. The flankers had one of five contrasts (10, 30, 50, 70, or 90%) and one of three SFs (1, 2, or 4 cycle/deg). Inner edges of flankers were 0.5° away from the screen center. The probe was a faint vertical line that appeared above or below the horizontal midline of the screen (as shown in Fig. 3, A and B) at one of five distances (0°, 0.125°, 0.25°, 0.375°, 0.4375°, or 0.5°, indicated in the abscissa of Fig. 3C) from one of the inner edges of the flankers. Probe contrast was controlled by an adaptive staircase procedure described just below. The task was to report whether the probe was seen in the upper or lower position.

#### Procedure

Each trial started with a fixation dot, which had a Gaussian luminance profile with the spatial constant of 0.17°, presented at screen center for 200 ms. Fixation was followed by the stimulus presented for 250 ms. Subjects used the upward and downward arrow keys on the keyboard to report whether they saw the probe appear in the upper or lower position. Subject’s response triggered the next trial. Contrast detection threshold of the probe was measured for each stimulus condition (spatial distance from the flanker, flanker SF, and flanker contrast) using a three-down, one-up adaptive staircase procedure. Starting with an initial contrast of 40%, probe contrast was either reduced, following three correct responses, or increased, following a single incorrect response. Contrast steps (same up and down) were 12% up to the second reversal of contrast, after which contrast steps were reduced to 3%. The procedure was terminated after 30 trials. Contrast threshold was estimated by averaging the last five values of contrast selected by the procedure in each stimulus condition. Experimental runs with the same stimulus conditions were repeated in each subject three to four times. Since we found no statistically significant differences between estimates of probe detection threshold at different flanker contrasts, the data presented in Fig. 3C are averaged across flanker contrasts. In effect, results of each subject are derived from data collected in 8100 to 10,800 trials. The data plotted in Fig. 3C were fitted by a damped harmonic function F(*d*) cos (2π*fd* + φ), where F(*d*) = Oexp ( − *d*^2^/(2*c*^2^)), *d* is the distance from the flanker, *f* is the SF, φ is the phase, O is the amplitude, and *c* is the damping coefficient.

### Modeling methods

#### Definition of the distributed E/I network

We have studied a system of equations for a chain of Wilson-Cowan motifs each containing an excitatory cell (*E*) and an inhibitory cell (*I*) as shown in Fig. 1A. The generic form of the system is

$$\tau_{E}\frac{dr_{E}(l)}{\mathrm{dt}}=-r_{E}+g_{E}(C_{E})$$

$$\frac{dr_{I}(l)}{\mathrm{dt}}=-r_{I}+g_{I}(C_{I})$$ (1)

where *r_E_* and *r_I_* represent the firing rates of the excitatory and inhibitory cells, τ*_E_* represents the relaxation time of excitation (in units of the relaxation time of inhibition), *l* is the location index in the chain, and *g_E_* and *g_I_* are sigmoid functions. The terms C*_E_* and C*_I_* represent sources of cell activation (from stimulation and from other cells in the network) at location *l*

$$\begin{array}{c} C_{E}=[w_{\mathrm{EE}}r_{E}(l)+{\tilde{w}}_{\mathrm{EE}}r_{E}(l+1)+{\tilde{w}}_{\mathrm{EE}}r_{E}(l-1)]\\ -[w_{\mathrm{EI}}r_{I}(l)+{\tilde{w}}_{\mathrm{EI}}r_{I}(l+1)+{\tilde{w}}_{\mathrm{EI}}r_{I}(l-1)]+i_{E}(l,t),\\ C_{I}=[w_{\mathrm{IE}}r_{E}(l)+{\tilde{w}}_{\mathrm{IE}}r_{E}(l+1)+{\tilde{w}}_{\mathrm{IE}}r_{E}(l-1)]\\ -[w_{\mathrm{II}}r_{I}(l)+{\tilde{w}}_{\mathrm{II}}r_{I}(l+1)+{\tilde{w}}_{\mathrm{II}}r_{I}(l-1)]+i_{I}(l,t) \end{array}$$

where *w* and $\tilde{w}$ represent the weights of connections within and between the motifs, respectively (*80*).

When modulations of neural activity occur on a spatial scale significantly larger than the distance between the nearest network motifs, and at stimulus contrasts for which the system response is far below saturation, the excitatory/inhibitory network can be modeled by a system of nonlinear partial differential equations (derived in the Supplementary Materials) of the form

$$\begin{array}{c} \tau_{E}\frac{\partial r_{E}}{\partial t}=-r_{E}+W_{\mathrm{EE}}r_{E}+D_{\mathrm{EE}}\frac{\partial^{2}r_{E}}{\partial x^{2}}-W_{\mathrm{EI}}r_{I}-D_{\mathrm{EI}}\frac{\partial^{2}r_{I}}{\partial x^{2}}\\ +\alpha j-\gamma_{E}{\left(\tau_{E}\frac{\partial r_{E}}{\partial t}+r_{E}\right)}^{3}\\ \frac{\partial r_{I}}{\partial t}=-r_{I}+W_{IE}r_{E}+D_{\mathrm{IE}}\frac{\partial^{2}r_{E}}{\partial x^{2}}-W_{\mathrm{II}}r_{I}-D_{\mathrm{II}}\frac{\partial^{2}r_{I}}{\partial x^{2}}\\ +(1-\alpha )j-\gamma_{I}{\left(\frac{\partial r_{I}}{\partial t}+r_{I}\right)}^{3} \end{array}$$ (2)

where *r_E_*(*x*, *t*) and *r_I_*(*x*, *t*) are firing rates of excitatory and inhibitory neurons at position *x* and time *t*, and γ*_E_* and γ*_I_* are Taylor expansion coefficients that arise due to nonlinearity of Wilson-Cowan sigmoid activation function [(*10*), p. 4]. The weights *W_EE_*, *W_EI_*, *W_IE_*, and*W_II_* control local interactions

$$W_{\mathrm{EE}}=w_{\mathrm{EE}}+2{\tilde{w}}_{\mathrm{EE}},W_{\mathrm{EI}}=w_{\mathrm{EI}}+2{\tilde{w}}_{\mathrm{EI}},\text{etc}.$$

The terms *D_EE_*, *D_EI_*, *D_IE_*, and *D_II_* are responsible for spatial spread of activation

$$D_{\mathrm{EE}}={\tilde{w}}_{\mathrm{EE}},D_{\mathrm{EI}}={\tilde{w}}_{\mathrm{EI}},\text{etc}.$$

The stimulus is represented by the input current *j*(*x*, *t*) divided between the excitatory and inhibitory neurons with fractions α and 1 − α such that the excitatory input is α*j* and the inhibitory input is (1 − α)*j*.

#### Intrinsic wave

##### Point activation

First, we considered that the system responds to a point stimulus *j*(*x*, *t*) = δ(*x*), where δ(*x*) is the Dirac delta function. The stimulus activates a singe motif of the chain. In linear approximation, the static solution has the form

$$\begin{array}{c} G_{E}=e^{-\lambda |x|}(\Gamma_{E}\text{cos}\;(k_{n}x)-\Delta_{E}\text{sign}(x)\text{sin}\;(k_{n}x))\\ G_{I}=e^{-\lambda |x|}(\Gamma_{I}\text{cos}\;(k_{n}x)-\Delta_{I}\text{sign}(x)\text{sin}\;(k_{n}x)) \end{array}$$ (3)

where sign(*x*) = *x*/∣*x*∣ for *x* ≠ 0 and sign(0) = 0. Equation 3 determines a spatial oscillation (Fig. 1C). The intrinsic SF *k*_n_, the rate of spatial decay λ, and parameters that determine the amplitude of oscillation (Γ*_E_*, Γ*_I_*, Δ*_E_*, and Δ*_I_*) are functions of the weights of neuronal connections *W_EE_*, *W_EI_*, *W_IE_*, *W_II_* and *D_EE_*, *D_EI_*, *D_IE_*, *D_II_*.

##### Distributed activation

For stimuli more complex than a delta function, the general static solution can be written in linear approximation as

$$\begin{array}{c} r_{E}=\int_{-\infty }^{\infty }j(x\prime )G_{E}(x-x\prime )dx\prime \\ r_{I}=\int_{-\infty }^{\infty }j(x\prime )G_{I}(x-x\prime )dx\prime \end{array}$$ (4)

suggesting how our model is related to the standard description of visual neural mechanisms in terms of linear filters. For example, for two point stimuli described by *j* = δ(*x* − *l*/2) + δ(*x* + *l*/2) separated by distance *l*, the excitatory response is defined simply as *r_E_* = *G_E_*(*x* − *l*/2) + *G_E_*(*x* + *l*/2) (Fig. 1D).

##### Induced activation

If the induced activity in zone 2 had a pure decaying profile outside the stimuli, such as *r_E_* = *A* exp (− λ(*x* + *x*_0_)) from stimulus located at *x* < − *x*_0_ and *r_E_* = *B* exp (− λ(*x*_1_ − *x*)) from the other stimulus located at *x* > *x*_1_, with spatial decay λ and activation *A* and *B* of excitatory cells generated by the corresponding stimuli at their boundaries, the combine activity from both stimuli

$$r_{E}=A\;\text{exp}\;(-\lambda (x+x_{0}))+B\;\text{exp}\;(-\lambda (x_{1}-x))$$

will produce no oscillations between *x* = − *x*_0_ and *x* = *x*_1_. By computing the spatial derivative of *r_E_* (to estimate possible positions of wave minima or maxima)

$$dr_{E}/\mathrm{dx}=-\lambda A\;\text{exp}\;(-\lambda (x+x_{0}))+\lambda B\;\text{exp}\;(-\lambda (x_{1}-x))=0$$

we obtain the only possible minimum of activity at

$$x_{\text{min}}=\text{ln}\;(A\;\text{exp}\;(\lambda (x_{1}-x_{0}))/B)/2\lambda$$

in contrast to a wave profile that is expected to have multiple minima. This result is consistent with the notion that the wave-shaped modulation of activity observed in our psychophysical study (Fig. 3) is produced by neural waves and that it cannot be explained by having the influence of the flanker stimulus merely decaying as a function of distance from the flanker in zone 2.

#### Linear resonance analysis

To understand the interaction of system responses to stimulus spatial (*k*) and temporal (ω) frequencies, we studied system response to input current *j*(*x*, *t*) produced by drifting luminance gratings *j* = *j*_0_ cos (*kx* − ω*t*) as stimuli. In linear approximation, the response of Eq. 2 to such spatiotemporally harmonic stimuli is

$$\begin{array}{c} r_{E}(x,t)={ℰ}_{C}\text{cos}\;(\mathrm{kx}-\omega t)+{ℰ}_{S}\text{sin}\;(\mathrm{kx}-\omega t)\\ r_{I}(x,t)={ℐ}_{C}\text{cos}\;(\mathrm{kx}-\omega t)+{ℐ}_{S}\text{sin}\;(\mathrm{kx}-\omega t) \end{array}$$ (5)

By substituting Eq. 5 in Eq. 2, we obtained algebraic equations for amplitudes ℰ_C_, ℰ_S_, ℐ_C_, and ℐ_S_. As in our nonlinear analysis of resonance behavior below (Eq. 13), the algebraic equations can be written in matrix form

$$ℋZ=I_{0}$$ (6)

where

$$H=[\begin{array}{cccc} W_{\mathrm{EE}}-1-k^{2}D_{\mathrm{EE}} & \tau_{E}\omega & -W_{\mathrm{EI}}+k^{2}D_{\mathrm{EI}} & 0\\ -\tau_{E}\omega & W_{\mathrm{EE}}-1-k^{2}D_{\mathrm{EE}} & 0 & -W_{\mathrm{EI}}+k^{2}D_{\mathrm{EI}}\\ W_{\mathrm{IE}}-k^{2}D_{\mathrm{IE}} & 0 & -W_{\mathrm{II}}-1+k^{2}D_{\mathrm{II}} & \omega \\ 0 & W_{\mathrm{IE}}-k^{2}D_{\mathrm{IE}} & -\omega & -W_{\mathrm{II}}-1+k^{2}D_{\mathrm{II}} \end{array}]$$

is the matrix of wave-component interaction, ***Z*** = (ℰ*_C_*, ℰ*_S_*, ℐ*_C_*, ℐ*_S_*)*^T^* is the wave-component vector, and ***I***_0_ = −*j*_0_(α,0,1 − α,0)*^T^* is the stimulation.

Elements of the matrix H describe the interaction of neural wave components. These elements depend on spatial and temporal stimulus frequencies, *k* and ω, as well as on coefficients of interaction between cells: local coefficients *W* and propagation coefficients *D*. In effect, spatial resonance depends on ω and temporal resonance depends on *k*, as we show below.

Using Cramer’s rule, the solution of Eq. 6 can be written as

$$Z=(\text{det}\;{ℋ}_{1},\text{det}\;{ℋ}_{2},\text{det}\;{ℋ}_{3},\text{det}\;{ℋ}_{4})/\text{det}\;ℋ$$ (7)

where ℋ*_i_* are standard matrices derived from ℋ by substituting its corresponding columns with ***I***_0_. We used Eq. 7 to derive Fig. 4.

To estimate stimulus frequencies at which the firing rate reaches its maximum (thus defining system selectivity), we assumed that the denominator *H* = det ℋ of the solution for ***Z*** has a corresponding minimum. In this approximation, the problem of finding the maximum of activity is reduced to the analysis of *H*, which has this form

$$\begin{array}{c} H=\underset{\text{Term A}}{\underbrace{\mu {\left[{\left(k^{2}-k_{n}^{2}+\lambda^{2}\right)}^{2}+4k_{n}^{2}\lambda^{2}\right]}^{2}}}+\underset{\text{Term B}}{\underbrace{\omega^{2}[\kappa_{4}k^{4}-\kappa_{2}k^{2}]}}\\ +\underset{\text{Term C}}{\underbrace{\omega^{2}[\tau_{E}^{2}\omega^{2}+\kappa_{0}]}} \end{array}$$ (8)

where μ, κ_4_, κ_2_, and κ_0_ are weight-dependent constants defined in the Supplementary Materials (eqs. S17 to S21). Note that Term A depends on *k* and not on ω, whereas Term C depends on ω and not on *k*. Accordingly, Term A describes the system’s spatial selectivity (at ω = 0), Term C describes the system’s temporal selectivity (at *k* = 0), and Term B, which contains products of ω and *k*, describes the interaction of temporal and spatial stimulus dimensions.

We used Eq. 8 to derive the spatial (Fig. 5A) and temporal (Fig. 5B) maxima of the system response by finding the minima of *H* at fixed ω and fixed *k*, respectively. In other words, the condition *∂H*/*∂k* = 0 is satisfied at the SFR *k*_r_(ω), and the condition *∂H*/∂ω = 0 is satisfied at the TFR ω_ctr_(*k*).

Using the equation *∂H*/*∂k* = 0, we obtain the frequency of spatial resonance

$$\omega^{2}=\frac{2\mu }{\kappa_{4}}\;\frac{\left[{\left(k_{r}^{2}-k_{n}^{2}+\lambda^{2}\right)}^{2}+4k_{n}^{2}\lambda^{2}](k_{r}^{2}-k_{n}^{2}+\lambda^{2}\right)}{\kappa_{\text{asymp}}-k_{r}^{2}}$$ (9)

where κ_asymp_ = κ_2_/2κ_4_. The vertical asymptote (displayed in Fig. 5, A and B) that separates two regimes of system behavior is given by the conditions in which the denominator of Eq. 9 is zero, i.e., at

$$k^{2}=\kappa_{\text{asymp}}=\frac{\left(W_{\mathrm{EE}}-1)D_{\mathrm{EE}}-\tau_{E}^{2}(W_{\mathrm{II}}+1)D_{\mathrm{II}}-\tau_{E}(W_{\mathrm{EI}}D_{\mathrm{IE}}+W_{\mathrm{IE}}D_{\mathrm{EI}}\right)}{D_{\mathrm{EE}}^{2}-2\tau_{E}D_{\mathrm{EI}}D_{\mathrm{IE}}+\tau_{E}^{2}D_{\mathrm{II}}^{2}}.$$

If $k_{n}^{2}-\lambda^{2}<\kappa_{\text{asymp}}$, then *k_r_* increases with ω from $k_{r}=\sqrt{k_{n}^{2}-\lambda^{2}}$ at ω = 0 to $k_{r}=\sqrt{\kappa_{\text{asymp}}}$ when ω → ∞; in the opposite case $k_{n}^{2}-\lambda^{2}>\kappa_{\text{asymp}}$, *k*_r_ decreases with ω from $k_{r}=\sqrt{k_{n}^{2}-\lambda^{2}}$ at ω = 0 to $k_{r}=\sqrt{\kappa_{\text{asymp}}}$ when ω → ∞.

By solving the equation *∂H*/∂ω = 0, we derive the expression for temporal resonance

$$\omega_{r}^{2}=\frac{1}{2\tau_{E}^{2}}(\kappa_{2}k^{2}-\kappa_{4}k^{4}-\kappa_{0})$$ (10)

#### Nonlinear resonance analysis

To study circuit behavior beyond linear approximation, and to investigate how resonance frequency of the circuit should change with contrast, we derived system response to spatially periodic stimuli of the form *j*(*x*) = *j*_0_ cos *kx*, where *k* is SF. By substituting into Eq. 2 an approximate solution in the form

$$r_{E}(x)=ℰ\;\text{cos}\;\mathrm{kx}$$ (11)

$$r_{I}(x)=ℐ\;\text{cos}\;\mathrm{kx}$$ (12)

while ignoring higher-order spatial harmonics (such as cos 3*kx* and sin 3*kx*), we obtained a nonlinear algebraic equation for the amplitudes of excitatory (ℰ) and inhibitory (ℐ) wave components. The result can be written in matrix form, as we did in Eq. 6

$$ℳY=J_{0}$$ (13)

where ***Y*** = (ℰ, ℐ)*^T^* is the wave-component vector, ***J***_0_ = − *j*_0_(α,1 − α)*^T^* is the stimulation, and ℳ is the matrix of the wave-component interaction

$$\left[\begin{array}{cc} M_{\mathrm{EE}} & M_{\mathrm{EI}}\\ M_{\mathrm{IE}} & M_{\mathrm{II}} \end{array}]=[\begin{array}{cc} W_{\mathrm{EE}}-\frac{3}{4}\gamma_{E}E^{2}-1-k^{2}D_{\mathrm{EE}} & -W_{\mathrm{EI}}+k^{2}D_{\mathrm{EI}}\\ W_{\mathrm{IE}}-k^{2}D_{\mathrm{IE}} & -W_{\mathrm{II}}-\frac{3}{4}\gamma_{I}I^{2}-1+k^{2}D_{\mathrm{II}} \end{array}\right]$$

The latter matrix represents mutual influence of wave components. Two terms of the matrix—M*_EE_* and M*_II_*—depend on the amplitudes E and ℐ, indicating that the interaction of wave components depends on stimulus contrast (in addition to its dependence on *k* and on weights of connections between cells). This dependence on the amplitudes of wave components is negligible at low contrasts (where terms that include γ*_E_* and γ*_I_* are very small), and it becomes progressively more prominent as ℰ and ℐ increase with contrast.

It is useful to distinguish between two kinds of coefficients describing circuit interactions. On the one hand, coefficients *W* and *D* in Eqs. 1 and 2 depend solely on weights of synaptic connections between cells (henceforth “cell interaction coefficients”) that describe stimulus-independent interaction of cells in the circuit. On the other hand, the terms M*_EE_*, M*_EI_*, M*_IE_*, and M*_II_* in Eq. 13 describe the interaction of components of neural waves rather than the interaction of cells. These coefficients M (henceforth “wave interaction coefficients”) depend both on cell interaction coefficients and on wave properties (such as wave frequency and amplitude). As a result, wave interaction coefficients depend on stimulus frequency and contrast.

To investigate effects of stimulus contrast, we rewrite Eq. 13 as

$$Y(J_{0})={ℳ}^{-1}(Y(J_{0}))J_{0}$$ (14)

where M^−1^(***Y***) is an inverse matrix. Equation 19 can be solved iteratively

$$Y_{n+1}={ℳ}^{-1}(Y_{n})J_{0}$$

where *n* is the iteration step number. The iterative procedure can be written explicitly

$$\begin{array}{c} {ℐ}_{n+1}=j_{0}\frac{\Psi_{E}+\Phi_{E}k^{2}+\eta_{I}{ℐ}_{n}^{2}}{{\left(k^{2}-k_{n}^{2}+\lambda^{2}+\xi_{I}{ℐ}_{n}^{2}-\xi_{E}{ℰ}_{n}^{2}\right)}^{2}+4\lambda^{2}k_{n}^{2}+\sigma_{I}{ℐ}_{n}^{2}-\sigma_{E}{ℰ}_{n}^{2}}\\ {ℰ}_{n+1}=j_{0}\frac{\Psi_{I}+\Phi_{I}k^{2}+\eta_{E}{ℰ}_{n}^{2}}{\underset{\text{Term A}}{\underbrace{{\left(k^{2}-k_{n}^{2}+\lambda^{2}+\xi_{I}{ℐ}_{n}^{2}-\xi_{E}{ℰ}_{n}^{2}\right)}^{2}}}+\underset{\text{Term B}}{\underbrace{4\lambda^{2}k_{n}^{2}+\sigma_{I}{ℐ}_{n}^{2}-\sigma_{E}{ℰ}_{n}^{2}}}} \end{array}$$ (15)

where constants Ψ*_E_*, Ψ*_I_*, Φ*_E_*, and Φ*_I_* are functions of cell interaction coefficients. Constants η*_E_*, η*_I_*, ξ*_E_*, ξ*_I_*, σ*_E_*, and σ*_I_* are also functions of cell interaction coefficients; they are proportional to the nonlinearity parameters γ*_E_* and γ*_I_*. A zero of Term A in the denominator of Eq. 15 corresponds to the condition of system resonance at zero TF (similar to the minimum of *H* in Eq. 8), and Term B in the denominator of Eq. 15 determines the extent of selectivity, which is the “width” of tuning to SF. Equation 15 allows one to derive results of the (*n* + 1)th iterations for E_*n* + 1_ and ℐ_*n* + 1_ using results of the *n*th iterations for E*_n_* and ℐ*_n_*. We used the iterative procedure defined by Eq. 15 to produce Fig. 8 (A to C).

Conditions of spatial resonance in the circuit, where Term A in the denominator of Eq. 15 is zero, are

$$k_{r}^{2}=(k_{n}^{2}-\lambda^{2})-\xi_{I}\;{ℐ}^{2}+\xi_{E}\;{ℰ}^{2}$$ (16)

The term $k_{n}^{2}-\lambda^{2}$ in Eq. 16 determines the resonance frequency *k*_r_ at low contrasts. Notice that *k*_r_ and *k_n_* could coincide when wave damping in the system is low (i.e., when λ is small). Notice also that the terms for E and ℐ in Eq. 16 have opposite signs. To appreciate this result, suppose that excitatory and inhibitory waves have similar magnitudes, ℰ = ℐ = A. We can therefore rewrite Eq. 16 as

$$k_{r}^{2}=(k_{n}^{2}-\lambda^{2})-A^{2}(\xi_{I}-\xi_{E})$$ (17)

Given that the terms $k_{n}^{2}-\lambda^{2}$, ξ*_I_*, and ξ*_E_* are constants, and that A is an increasing function of contrast, resonance frequency *k_r_* will decrease with contrast when ξ*_I_* > ξ*_E_* (Fig. 8C) and increase with contrast when ξ*_I_* < ξ*_E_* (Fig. 8B).

## Supplementary Materials

### This PDF file includes:

Figs. S1 to S5 Supplementary Modeling Methods

## Notes

View/request a protocol for this paper from *Bio-protocol*.

## References

[1] R. A. Holub, M. Morton-Gibson, Response of visual cortical neurons of the cat to moving sinusoidal gratings: Response-contrast functions and spatiotemporal interactions. J. Neurophysiol. 46, 1244–1259 (1981). 7320745 10.1152/jn.1981.46.6.1244

[2] J. Allman, F. Miezin, E. McGuinness, Stimulus specific responses from beyond the classical receptive field: Neurophysiological mechanisms for local-global comparisons in visual neurons. Annu. Rev. Neurosci. 8, 407–430 (1985). 3885829 10.1146/annurev.ne.08.030185.002203

[3] G. C. DeAngelis, R. D. Freeman, I. Ohzawa, Length and width tuning of neurons in the cat’s primary visual cortex. J. Neurophysiol. 71, 347–374 (1994). 8158236 10.1152/jn.1994.71.1.347

[4] D. G. Albrecht, Visual cortex neurons in monkey and cat: Effect of contrast on the spatial and temporal phase transfer functions. Vis. Neurosci. 12, 1191–1210 (1995). 8962836 10.1017/s0952523800006817

[5] T. D. Albright, G. R. Stoner, Contextual influences on visual processing. Annu. Rev. Neurosci. 25, 339–379 (2002). 12052913 10.1146/annurev.neuro.25.112701.142900

[6] M. P. Sceniak, M. J. Hawken, R. Shapley, Contrast-dependent changes in spatial frequency tuning of macaque V1 neurons: Effects of a changing receptive field size. J. Neurophysiol. 88, 1363–1373 (2002). 12205157 10.1152/jn.2002.88.3.1363

[7] N. J. Priebe, S. G. Lisberger, J. A. Movshon, Tuning for spatiotemporal frequency and speed in directionally selective neurons of macaque striate cortex. J. Neurosci. 26, 2941–2950 (2006). 16540571 10.1523/JNEUROSCI.3936-05.2006 PMC2532672

[8] D. B. Rubin, S. D. Van Hooser, K. D. Miller, The stabilized supralinear network: A unifying circuit motif underlying multi-input integration in sensory cortex. Neuron 85, 402–417 (2015). 25611511 10.1016/j.neuron.2014.12.026 PMC4344127

[9] A. S. Pawar, S. Gepshtein, S. Savel’ev, T. D. Albright, Mechanisms of spatiotemporal selectivity in cortical area MT. Neuron 101, 514–527.e2 (2019). 30606614 10.1016/j.neuron.2018.12.002 PMC6398985

[10] H. R. Wilson, J. D. Cowan, Excitatory and inhibitory interactions in localized populations of model neurons. Biophys. J. 12, 1–24 (1972). 4332108 10.1016/S0006-3495(72)86068-5 PMC1484078

[11] H. R. Wilson, J. D. Cowan, A mathematical theory of the functional dynamics of cortical and thalamic nervous tissue. Kybernetik 13, 55–80 (1973). 4767470 10.1007/BF00288786

[12] J. Rinzel, D. Terman, X.-J. Wang, B. Ermentrout, Propagating activity patterns in large-scale inhibitory neuronal networks. Science 279, 1351–1355 (1998). 9478895 10.1126/science.279.5355.1351

[13] S. Coombes, G. J. Lord, M. R. Owen, Waves and bumps in neuronal networks with axo-dendritic synaptic interactions. Phys. Nonlinear Phenom. 178, 219–241 (2003).

[14] P. A. Robinson, X. Zhao, K. M. Aquino, J. D. Griffiths, S. Sarkar, G. Mehta-Pandejee, Eigenmodes of brain activity: Neural field theory predictions and comparison with experiment. Neuroimage 142, 79–98 (2016). 27157788 10.1016/j.neuroimage.2016.04.050

[15] S. Heitmann, G. B. Ermentrout, Direction-selective motion discrimination by traveling waves in visual cortex. PLOS Comput. Biol. 16, e1008164 (2020). 32877405 10.1371/journal.pcbi.1008164 PMC7467221

[16] P. Gong, C. van Leeuwen, Distributed dynamical computation in neural circuits with propagating coherent activity patterns. PLOS Comput. Biol. 5, e1000611 (2009). 20019807 10.1371/journal.pcbi.1000611 PMC2787923

[17] E. Salinas, T. J. Sejnowski, Correlated neuronal activity and the flow of neural information. Nat. Rev. Neurosci. 2, 539–550 (2001). 11483997 10.1038/35086012 PMC2868968

[18] C. C. Petersen, A. Grinvald, B. Sakmann, Spatiotemporal dynamics of sensory responses in layer 2/3 of rat barrel cortex measured in vivo by voltage-sensitive dye imaging combined with whole-cell voltage recordings and neuron reconstructions. J. Neurosci. 23, 1298–1309 (2003). 12598618 10.1523/JNEUROSCI.23-04-01298.2003 PMC6742278

[19] M. P. Jadi, T. J. Sejnowski, Regulating cortical oscillations in an inhibition-stabilized network. Proc. IEEE 102, 830–842 (2014). 10.1109/JPROC.2014.2313113 PMC4067313 24966414

[20] J. F. Mejias, J. D. Murray, H. Kennedy, X.-J. Wang, Feedforward and feedback frequency-dependent interactions in a large-scale laminar network of the primate cortex. Sci. Adv. 2, e1601335 (2016). 28138530 10.1126/sciadv.1601335 PMC5262462

[21] W.-J. Song, H. Kawaguchi, S. Totoki, Y. Inoue, T. Katura, S. Maeda, S. Inagaki, H. Shirasawa, M. Nishimura, Cortical intrinsic circuits can support activity propagation through an isofrequency strip of the guinea pig primary auditory cortex. Cereb. Cortex 16, 718–729 (2006). 16107586 10.1093/cercor/bhj018

[22] A. Benucci, R. A. Frazor, M. Carandini, Standing waves and traveling waves distinguish two circuits in visual cortex. Neuron 55, 103–117 (2007). 17610820 10.1016/j.neuron.2007.06.017 PMC2171365

[23] W. Xu, X. Huang, K. Takagaki, J. Wu, Compression and reflection of visually evoked cortical waves. Neuron 55, 119–129 (2007). 17610821 10.1016/j.neuron.2007.06.016 PMC1988694

[24] L. Muller, A. Reynaud, F. Chavane, A. Destexhe, The stimulus-evoked population response in visual cortex of awake monkey is a propagating wave. Nat. Commun. 5, 3675 (2014). 24770473 10.1038/ncomms4675 PMC4015334

[25] T. P. Zanos, P. J. Mineault, K. T. Nasiotis, D. Guitton, C. C. Pack, A sensorimotor role for traveling waves in primate visual cortex. Neuron 85, 615–627 (2015). 25600124 10.1016/j.neuron.2014.12.043

[26] M. V. Tsodyks, W. E. Skaggs, T. J. Sejnowski, B. L. McNaughton, Paradoxical effects of external modulation of inhibitory interneurons. J. Neurosci. 17, 4382–4388 (1997). 9151754 10.1523/JNEUROSCI.17-11-04382.1997 PMC6573545

[27] H. R. Wilson, *Spikes, Decisions, and Actions: The Dynamical Foundations of Neuroscience* (Oxford Univ. Press, 1999).

[28] A. F. Teich, N. Qian, Learning and adaptation in a recurrent model of V1 orientation selectivity. J. Neurophysiol. 89, 2086–2100 (2003). 12611961 10.1152/jn.00970.2002

[29] H. Ozeki, I. M. Finn, E. S. Schaffer, K. D. Miller, D. Ferster, Inhibitory stabilization of the cortical network underlies visual surround suppression. Neuron 62, 578–592 (2009). 19477158 10.1016/j.neuron.2009.03.028 PMC2691725

[30] Y. Ahmadian, D. B. Rubin, K. D. Miller, Analysis of the stabilized supralinear network. Neural Comput. 25, 1994–2037 (2013). 23663149 10.1162/NECO_a_00472 PMC4026108

[31] K. D. Miller, Canonical computations of cerebral cortex. Curr. Opin. Neurobiol. 37, 75–84 (2016). 26868041 10.1016/j.conb.2016.01.008 PMC4944655

[32] U. Polat, D. Sagi, Lateral interactions between spatial channels: Suppression and facilitation revealed by lateral masking experiments. Vision Res. 33, 993–999 (1993). 8506641 10.1016/0042-6989(93)90081-7

[33] I. Kovacs, B. Julesz, Perceptual sensitivity maps within globally defined visual shapes. Nature 370, 644–646 (1994). 8065449 10.1038/370644a0

[34] V. Manahilov, Triphasic temporal impulse responses and Mach bands in time. Vision Res. 38, 447–458 (1998). 9536368 10.1016/s0042-6989(97)00149-1

[35] D. J. Field, A. Hayes, R. F. Hess, Contour integration by the human visual system: Evidence for a local association field. Vision Res. 33, 173–193 (1993). 8447091 10.1016/0042-6989(93)90156-q

[36] C. D. Gilbert, T. N. Wiesel, Columnar specificity of intrinsic horizontal and corticocortical connections in cat visual cortex. J. Neurosci. 9, 2432–2442 (1989). 2746337 10.1523/JNEUROSCI.09-07-02432.1989 PMC6569760

[37] D. D. Stettler, A. Das, J. Bennett, C. D. Gilbert, Lateral connectivity and contextual interactions in macaque primary visual cortex. Neuron 36, 739–750 (2002). 12441061 10.1016/s0896-6273(02)01029-2

[38] J. D. Semedo, A. Zandvakili, C. K. Machens, B. M. Yu, A. Kohn, Cortical areas interact through a communication subspace. Neuron 102, 249–259.e4 (2019). 30770252 10.1016/j.neuron.2019.01.026 PMC6449210

[39] V. Manahilov, Spatiotemporal visual response to suprathreshold stimuli. Vision Res. 35, 227–237 (1995). 7839618 10.1016/0042-6989(94)00120-b

[40] J. J. Pattadkal, G. Mato, C. van Vreeswijk, N. J. Priebe, D. Hansel, Emergent orientation selectivity from random networks in mouse visual cortex. Cell Rep. 24, 2042–2050.e6 (2018). 30134166 10.1016/j.celrep.2018.07.054 PMC6179374

[41] N. Ju, Y. Li, F. Liu, H. Jiang, S. L. Macknik, S. Martinez-Conde, S. Tang, Spatiotemporal functional organization of excitatory synaptic inputs onto macaque V1 neurons. Nat. Commun. 11, 697 (2020). 32019929 10.1038/s41467-020-14501-y PMC7000673

[42] R. F. Hess, A. Hayes, D. J. Field, Contour integration and cortical processing. J. Physiol.-Paris 97, 105–119 (2003). 14766137 10.1016/j.jphysparis.2003.09.013

[43] D. A. Mély, D. Linsley, T. Serre, Complementary surrounds explain diverse contextual phenomena across visual modalities. Psychol. Rev. 125, 769–784 (2018). 30234321 10.1037/rev0000109

[44] H. R. Wilson, Binocular contrast, stereopsis, and rivalry: Toward a dynamical synthesis. Vision Res. 140, 89–95 (2017). 28882755 10.1016/j.visres.2017.07.016

[45] G. Riesen, A. M. Norcia, J. L. Gardner, Humans perceive binocular rivalry and fusion in a tristable dynamic state. J. Neurosci. 39, 8527–8537 (2019). 31519817 10.1523/JNEUROSCI.0713-19.2019 PMC6807276

[46] D. Tadin, J. S. Lappin, L. A. Gilroy, R. Blake, Perceptual consequences of centre–surround antagonism in visual motion processing. Nature 424, 312–315 (2003). 12867982 10.1038/nature01800

[47] D. Tadin, Suppressive mechanisms in visual motion processing: From perception to intelligence. Vision Res. 115, 58–70 (2015). 26299386 10.1016/j.visres.2015.08.005 PMC4587336

[48] D. M. Alexander, T. Ball, A. Schulze-Bonhage, C. van Leeuwen, Large-scale cortical travelling waves predict localized future cortical signals. PLOS Comput. Biol. 15, e1007316 (2019). 31730613 10.1371/journal.pcbi.1007316 PMC6894364

[49] H. Hogendoorn, Motion extrapolation in visual processing: Lessons from 25 years of flash-lag debate. J. Neurosci. 40, 5698–5705 (2020). 32699152 10.1523/JNEUROSCI.0275-20.2020 PMC7380963

[50] I. Kovacs, B. Julesz, A closed curve is much more than an incomplete one: Effect of closure in figure-ground segmentation. Proc. Natl. Acad. Sci. U.S.A. 90, 7495–7497 (1993). 8356044 10.1073/pnas.90.16.7495 PMC47168

[51] D. Tadin, J. S. Lappin, Optimal size for perceiving motion decreases with contrast. Vision Res. 45, 2059–2064 (2005). 15845238 10.1016/j.visres.2005.01.029

[52] R. T. Born, D. C. Bradley, Structure and function of visual area MT. Annu. Rev. Neurosci. 28, 157–189 (2005). 16022593 10.1146/annurev.neuro.26.041002.131052

[53] A. Angelucci, M. Bijanzadeh, L. Nurminen, F. Federer, S. Merlin, P. C. Bressloff, Circuits and mechanisms for surround modulation in visual cortex. Annu. Rev. Neurosci. 40, 425–451 (2017). 28471714 10.1146/annurev-neuro-072116-031418 PMC5697758

[54] D. H. Hubel, T. N. Wiesel, Receptive fields and functional architecture of monkey striate cortex. J. Physiol. 195, 215–243 (1968). 4966457 10.1113/jphysiol.1968.sp008455 PMC1557912

[55] R. Shapley, P. Lennie, Spatial frequency analysis in the visual system. Annu. Rev. Neurosci. 8, 547–583 (1985). 3920946 10.1146/annurev.ne.08.030185.002555

[56] J. P. Jones, L. A. Palmer, An evaluation of the two-dimensional Gabor filter model of simple receptive fields in cat striate cortex. J. Neurophysiol. 58, 1233–1258 (1987). 3437332 10.1152/jn.1987.58.6.1233

[57] R. L. De Valois, K. K. De Valois, *Spatial Vision* (Oxford Univ. Press, 1990).

[58] H. R. Wilson, J. R. Bergen, A four mechanism model for threshold spatial vision. Vision Res. 19, 19–32 (1979). 419698 10.1016/0042-6989(79)90117-2

[59] D. Marr, *Vision: A Computational Investigation Into the Human Representation and Processing of Visual Information* (W. H. Freeman and Company, 1982).

[60] E. H. Adelson, J. R. Bergen, Spatiotemporal energy models for the perception of motion. J. Opt. Soc. Am. A 2, 284–299 (1985). 3973762 10.1364/josaa.2.000284

[61] L. Muller, F. Chavane, J. Reynolds, T. J. Sejnowski, Cortical travelling waves: Mechanisms and computational principles. Nat. Rev. Neurosci. 19, 255–268 (2018). 29563572 10.1038/nrn.2018.20 PMC5933075

[62] Z. W. Davis, L. Muller, J. Martinez-Trujillo, T. Sejnowski, J. H. Reynolds, Spontaneous travelling cortical waves gate perception in behaving primates. Nature 587, 432–436 (2020). 33029013 10.1038/s41586-020-2802-y PMC7677221

[63] D. Fitzpatrick, The functional organization of local circuits in visual cortex: Insights from the study of tree shrew striate cortex. Cereb. Cortex 6, 329–341 (1996). 8670661 10.1093/cercor/6.3.329

[64] J. R. Cavanaugh, W. Bair, J. A. Movshon, Nature and interaction of signals from the receptive field center and surround in macaque V1 neurons. J. Neurophysiol. 88, 2530–2546 (2002). 12424292 10.1152/jn.00692.2001

[65] J. B. Levitt, J. S. Lund, in *Cortical Areas* (CRC Press, 2002), pp. 145–166.

[66] W. Bair, Visual receptive field organization. Curr. Opin. Neurobiol. 15, 459–464 (2005). 16023850 10.1016/j.conb.2005.07.006

[67] O. Schwartz, A. Hsu, P. Dayan, Space and time in visual context. Nat. Rev. Neurosci. 8, 522–535 (2007). 17585305 10.1038/nrn2155

[68] D. L. Ringach, The geometry of masking in neural populations. Nat. Commun. 10, 4879 (2019). 31653855 10.1038/s41467-019-12881-4 PMC6814762

[69] J. A. Movshon, W. T. Newsome, Visual response properties of striate cortical neurons projecting to area MT in macaque monkeys. J. Neurosci. 16, 7733–7741 (1996). 8922429 10.1523/JNEUROSCI.16-23-07733.1996 PMC6579106

[70] F. Zangeneh-Nejad, D. L. Sounas, A. Alù, R. Fleury, Analogue computing with metamaterials. Nat. Rev. Mater. 6, 207–225 (2021).

[71] G. Csaba, W. Porod, Coupled oscillators for computing: A review and perspective. Appl. Phys. Rev. 7, 011302 (2020).

[72] D. V. Buonomano, W. Maass, State-dependent computations: Spatiotemporal processing in cortical networks. Nat. Rev. Neurosci. 10, 113–125 (2009). 19145235 10.1038/nrn2558

[73] G. Tanaka, T. Yamane, J. B. Héroux, R. Nakane, N. Kanazawa, S. Takeda, H. Numata, D. Nakano, A. Hirose, Recent advances in physical reservoir computing: A review. Neural Netw. 115, 100–123 (2019). 30981085 10.1016/j.neunet.2019.03.005

[74] W. Wang, G. Pedretti, V. Milo, R. Carboni, A. Calderoni, N. Ramaswamy, A. S. Spinelli, D. Ielmini, Learning of spatiotemporal patterns in a spiking neural network with resistive switching synapses. Sci. Adv. 4, eaat4752 (2018). 30214936 10.1126/sciadv.aat4752 PMC6135543

[75] J. D. Monaco, K. Rajan, G. M. Hwang, A brain basis of dynamical intelligence for AI and computational neuroscience. arXiv:210507284 [q-bio.NC] (15 May 2021).

[76] J. Baladron, D. Fasoli, O. Faugeras, J. Touboul, Mean-field description and propagation of chaos in networks of Hodgkin-Huxley and FitzHugh-Nagumo neurons. J. Math. Neurosci. 2, 10 (2012). 22657695 10.1186/2190-8567-2-10 PMC3497713

[77] J. Senk, K. Korvasová, J. Schuecker, E. Hagen, T. Tetzlaff, M. Diesmann, M. Helias, Conditions for wave trains in spiking neural networks. Phys. Rev. Res. 2, 023174 (2020).

[78] J. M. Hutchinson, A. W. Lo, T. Poggio, A nonparametric approach to pricing and hedging derivative securities via learning networks. J. Finance 49, 851–889 (1994).

[79] X. Li, Z.-L. Lu, P. Xu, J. Jin, Y. Zhou, Generating high gray-level resolution monochrome displays with conventional computer graphics cards and color monitors. J. Neurosci. Methods 130, 9–18 (2003). 14583400 10.1016/s0165-0270(03)00174-2

[80] S. Savel’ev, S. Gepshtein, Neural wave interference in inhibition-stabilized networks, in *Proceedings of the Fist International Electronic Conference on Entropy and Its Applications*, D. Gencaga, Ed. (2014), p. c002, MDPI: Basel, Switzerland, doi:10.3390/ecea-1-c002.

[81] K. R. Dobkins, T. D. Albright, What happens if it changes color when it moves?: The nature of chromatic input to macaque visual area MT. J. Neurosci. 14, 4854–4870 (1994). 8046456 10.1523/JNEUROSCI.14-08-04854.1994 PMC6577181

