# Supplementary material for: Spatially distributed computation in cortical circuits
Source: Sci Adv. 2022 Apr 22;8(16):eabl5865. doi: 10.1126/sciadv.abl5865 (PMC9032974; doi:10.1126/sciadv.abl5865)
Supplement: Supplementary file 1 — Figs. S1 to S5 Supplementary Modeling Methods [file sciadv.abl5865_sm.pdf]

Science Advances
AAAS

Supplementary Materials for

Spatially distributed computation in cortical circuits

Sergei Gepshtein*, Ambarish S. Pawar, Sunwoo Kwon, Sergey Savel'ev*, Thomas D. Albright

*Corresponding author. Email: sergei@salk.edu (S.G.); s.saveliev@lboro.ac.uk (S.S.)

Published 22 April 2022, *Sci. Adv.* **8**, eabl5865 (2022)
DOI: 10.1126/sciadv.abl5865

This PDF file includes:

Figs. S1 to S5
Supplementary Modeling Methods

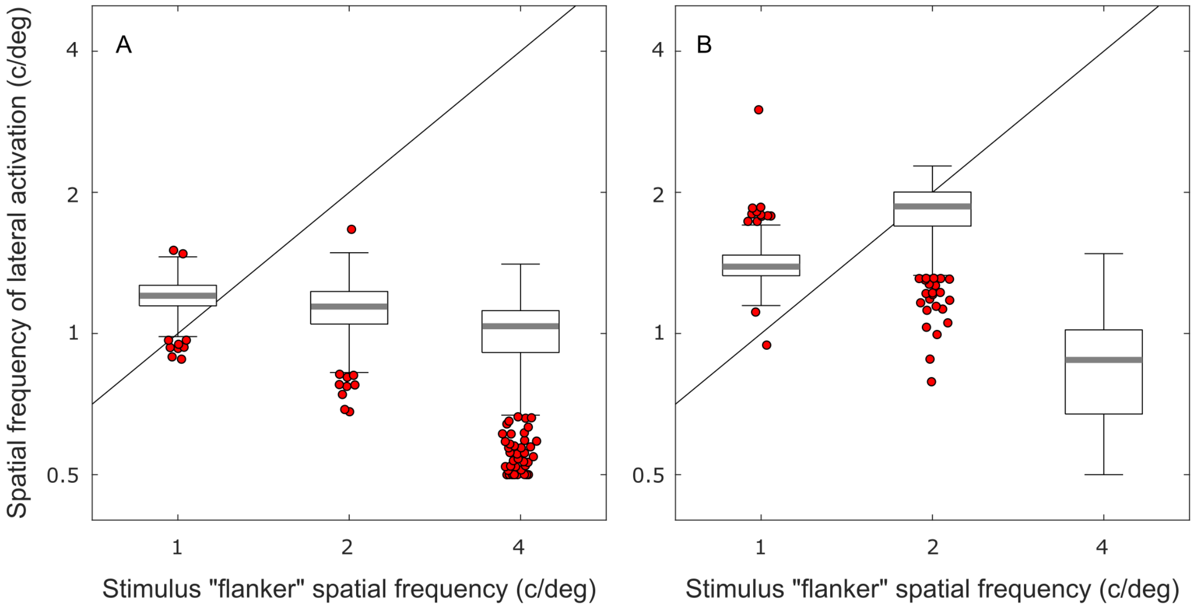

Supplemental Figure 1. Resampling analysis of spatial frequencies of lateral activation in human subjects

The boxplots represent results of resampling analysis of spatial frequencies (SF) of lateral activation waveforms fitted to contrast sensitivity data for two human subjects (Figure 3C). Results are shown for Subject 1 in panel A and for Subject 2 in panel B. Three values of stimulus SF in the abscissa of each panel correspond to three flanker SFs displayed on top of each column of plots in Figure 3C. The black line in each plot represents the SF of lateral activation that would have been observed if lateral activation had the same SF as stimulus SF.

Estimates of contrast sensitivity for each flanker SF were resampled with replacement 500 times. On each iteration of resampling, a harmonic function tapered by an exponential envelope was fitted to contrast sensitivity estimates for six probe-to-flanker distances (displayed in Figure 3C). Each boxplot represents a distribution of estimates of the SF of lateral activation: the horizontal gray bar marks the median, the top and bottom edges of the box mark the interquartile range (IQR), the whiskers mark the range of 1.5×IQR, and red dots represent the outliers.

For Subject 1, differences between estimates of lateral SF obtained at different values of stimulus SF were statistically insignificant (p=0.34 for stimulus SFs of 1 and 2 c/deg; p=0.26 for stimulus SFs of 2 and 4 c/deg). For Subject 2 the difference between estimates of lateral SF was statistically insignificant for stimulus SFs of 1 and 2 c/deg (p=0.07), yet lateral SF decreased significantly from stimulus SFs of 2 to stimulus SFs of 4 c/deg ($p \ll 0.01$). Parameters of the waveforms displayed in Figure 3C were obtained by averaging results of resampling.

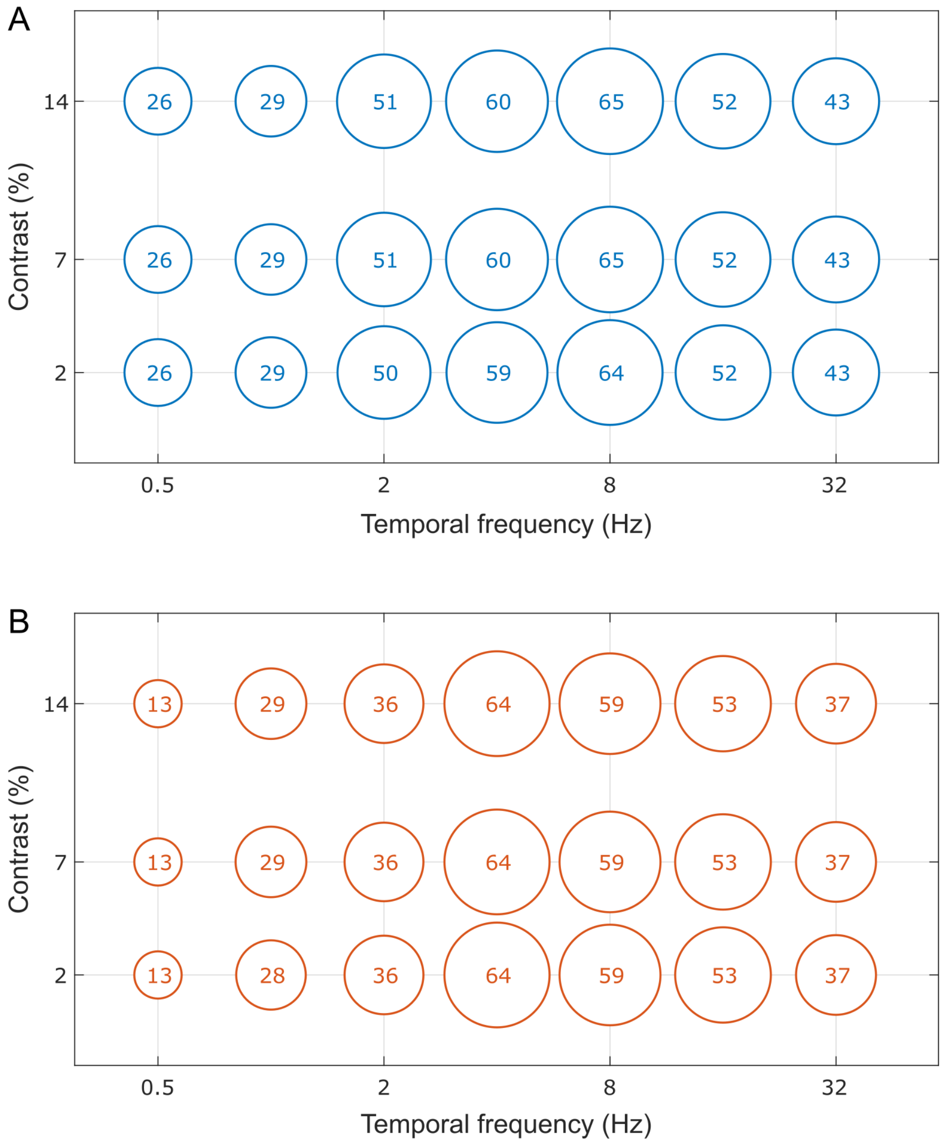

Supplemental Figure 2. Numbers of MT neurons measured to construct Figure 6

Circle sizes represent numbers of neurons measured in populations of MT neurons in Monkey 1 (A) and Monkey 2 (B) to obtain temporal response functions, such as those shown in the top row of plots in Figure 6. Each temporal response function was measured at seven stimulus temporal frequencies, separately for three stimulus contrasts (shown in this figure) and eight stimulus spatial frequencies (not shown here since there was little variation of neuron counts across stimulus spatial frequencies).

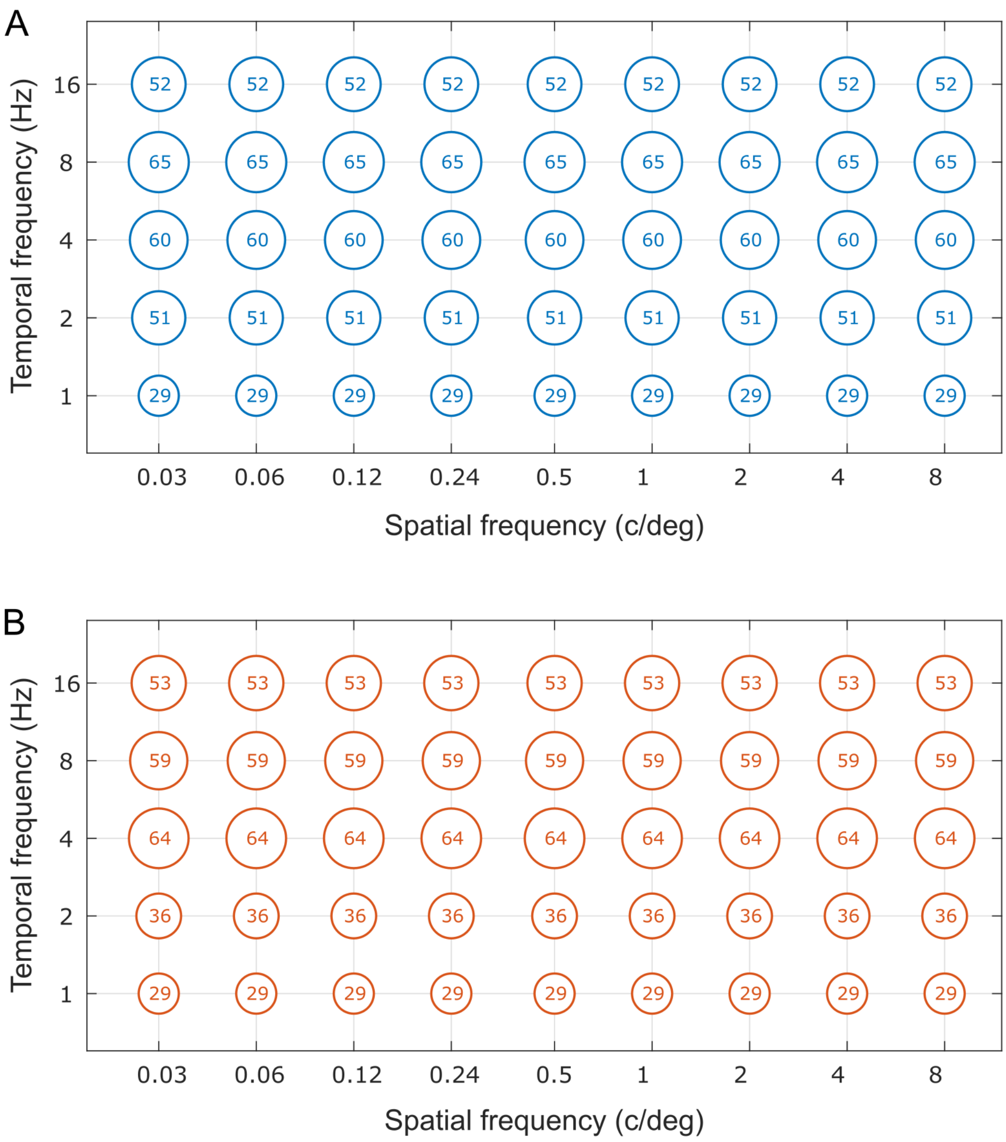

Supplemental Figure 3. Numbers of MT neurons measured to construct Figure 7

Circle sizes represent numbers of neurons measured in populations of MT neurons in Monkey 1 (A) and Monkey 2 (B) to obtain spatial response functions, such as those shown in the left column of plots in Figure 7. Each spatial response function was measured at nine stimulus spatial frequencies, separately for five stimulus temporal frequencies (shown in this figure) and three stimulus contrasts (not shown here since neuron counts for different stimulus contrasts were the same).

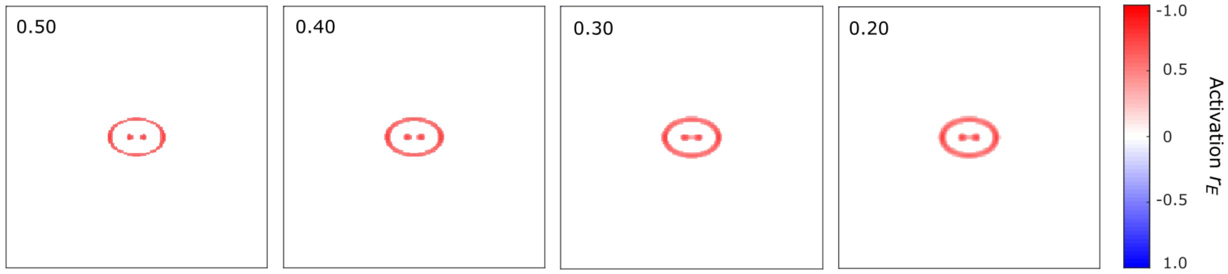

Supplemental Figure 4. The shape of the region of reduced behavioral threshold

The shape of the region of reduced threshold discovered by behavioral methods ("behavioral threshold") depends on the "neural threshold," which is the amount of neural activity required for stimulus detection. Here the shape of the region of reduced behavioral threshold is plotted for different assumed magnitudes of neural threshold (displayed in arbitrary units at top left of every panel). The lower the magnitude of neural threshold, the lower the neural activity sufficient for stimulus detection. For neural thresholds used in this illustration, only the central part of the response area contains detectable activation. Predicted regions of reduced behavioral threshold could have two separate foci in the center of the response area, as in two panels at left, or the foci could be connected, as in two panels at right. The same graphic conventions are used in this figure as in Figure 9B in the main text.

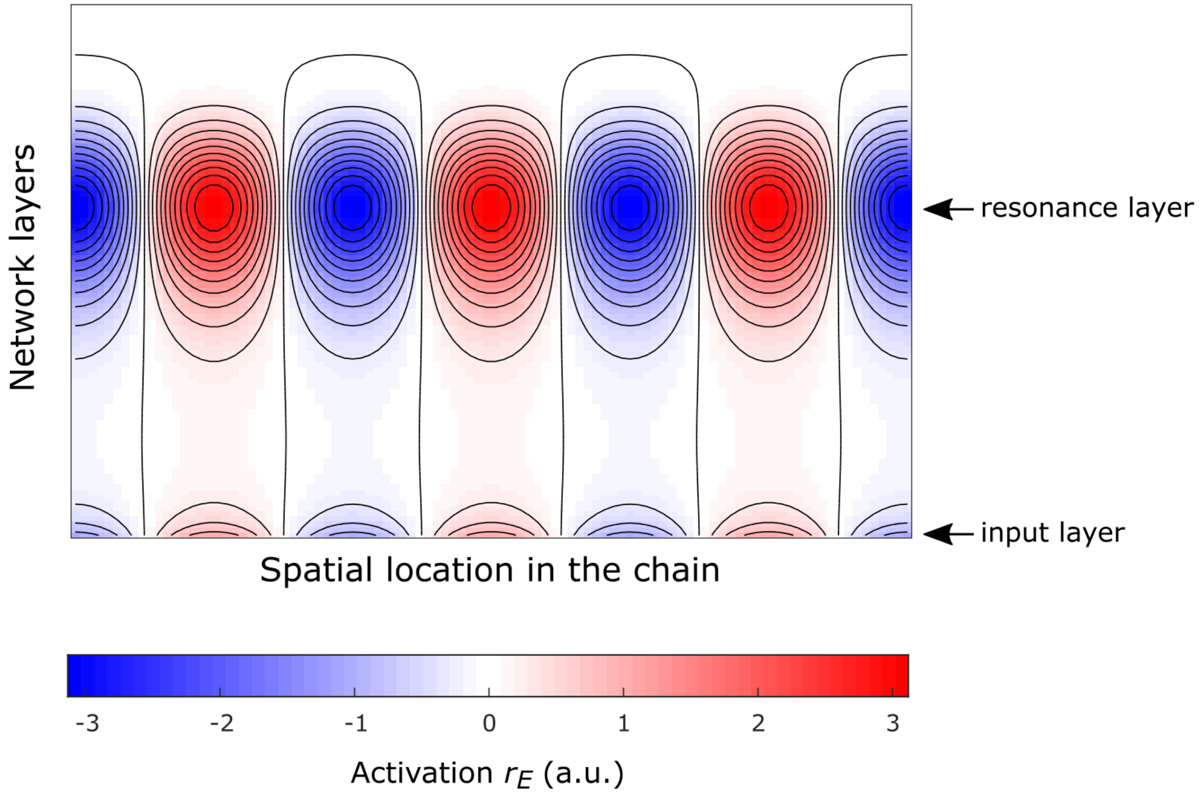

Supplemental Figure 5. Propagation of activity in a layered network

In the section “Propagation of activity in a layered network” of Supplemental Materials, we consider a variant of the distributed network, in which the one-dimensional chain shown in Figure 1B of the main text is replicated to form a series of parallel layers that represent stages of information processing in the nervous system, including “early” cortical areas. The layers are connected so that activity can propagate from one layer to another. Our analysis has demonstrated that neural waves can propagate between the layers, while the shape of the stimulus applied to the input layer is preserved (Supplemental Figure 5), and the amplitude of the wave is proportional to stimulus contrast. The plot represents results of numerical integration of Supplemental Equation 11, following the same graphic conventions as in Figure 1D in the main text. The contour lines represent levels of constant activation.

Supplemental Modeling Methods

Continuous model of spatially distributed Wilson-Cowan circuit

Consider a system of Wilson-Cowan equations for a chain of identical network motifs, each containing an excitatory cell ("E") and an inhibitory cell ("I"), as shown in Figure 1A. This network architecture is described by the following system of equations:

$$\tau_E \frac{dr_E(l)}{dt} = -r_E + g_E(\mathcal{C}_E),$$
$$\frac{dr_I(l)}{dt} = -r_I + g_I(\mathcal{C}_I). \tag{S1}$$

The variables r_E and r_I represent the firing rates of the excitatory and inhibitory cells, τ_E represents the relaxation time of the excitatory cell (in units of the relaxation time of the inhibitory cell), l is a discrete index of location in the chain, and g_E, g_I are sigmoid functions. The terms $\mathcal{C}_E$ and $\mathcal{C}_I$ represent sources of cell activation at each location:

$$\mathcal{C}_E = \Big[w_{EE}r_E(l) + \tilde{w}_{EE}r_E(l+1) + \tilde{w}_{EE}r_E(l-1)\Big] - \Big[w_{EI}r_I(l) + \tilde{w}_{EI}r_I(l+1) + \tilde{w}_{EI}r_I(l-1)\Big] + i_E(l,t),$$

$$\mathcal{C}_I = \Big[w_{IE}r_E(l) + \tilde{w}_{IE}r_E(l+1) + \tilde{w}_{IE}r_E(l-1)\Big] - \Big[w_{II}r_I(l) + \tilde{w}_{II}r_I(l+1) + \tilde{w}_{II}r_I(l-1)\Big] + i_I(l,t),$$

where w and $\tilde{w}$ represent the weights of connections respectively within and between the motif. We assume a fully interconnected nearest-neighbor network. That is, each inhibitory (excitatory) cell is connected to the excitatory (inhibitory) cell within motifs, and it is also connected to inhibitory and excitatory cells in the nearest motifs. The inter-motif connections allow activation to propagate through the chain.

We model neural activity in terms of spiking rates, r_E and r_I, using the Wilson-Cowan framework (S1), rather than modeling spiking directly, because the time scales of spike duration and inter-spike intervals are much shorter than the characteristic time of visual stimuli considered here. Future studies, concerned with higher-frequency stimuli, can use a more general framework that includes a model of spiking, as in the ongoing investigation of the relationship between spiking and mean activity of the network (Baladron et al., 2012; Senk et al., 2020). We assume that neuronal connections do not vary across location. We also assume that stimulus inputs i_E, i_I and cell responses r_E, r_I vary slowly on the scale of inter-motif distance, so that the spatial period of the stimulus is much larger than the inter-motif distance. For this case, we can replace the discrete index of chain location l by a continuous spatial variable x, to obtain

$$r_E(l \pm 1) = r_E(l) \pm \frac{\partial r_E}{\partial x} + \frac{1}{2}\frac{\partial^2 r_E}{\partial x^2},$$

$$r_I(l \pm 1) = r_I(l) \pm \frac{\partial r_I}{\partial x} + \frac{1}{2}\frac{\partial^2 r_I}{\partial x^2}.$$

We consider the conditions of stimulation in which the response does not approach the saturation value of the sigmoid activation function, so that only weak deviations from the linear response are possible. In this case, by expanding the inverse sigmoid functions g_E^{-1} and g_I^{-1} in the Taylor series up to the third order, we obtain from the above the following pair of coupled partial differential equations:

$$\begin{aligned} &W_{EE} r_E(x,t) + D_{EE}\frac{\partial^2 r_E}{\partial x^2} - W_{EI} r_I(x,t) - D_{EI}\frac{\partial^2 r_I}{\partial x^2} + \alpha j(x,t) \\ &= g_E^{-1}\left(\tau_E \frac{\partial r_E}{\partial t} + r_E\right) \approx \left(\tau_E \frac{\partial r_E}{\partial t} + r_E\right) + \beta_E \left(\tau_E \frac{\partial r_E}{\partial t} + r_E\right)^2 + \gamma_E \left(\tau_E \frac{\partial r_E}{\partial t} + r_E\right)^3, \\ &W_{IE} r_E(x,t) + D_{IE}\frac{\partial^2 r_E}{\partial x^2} - W_{II} r_I(x,t) - D_{II}\frac{\partial^2 r_I}{\partial x^2} + (1-\alpha) j(x,t) \\ &= g_I^{-1}\left(\frac{\partial r_I}{\partial t} + r_I\right) \approx \left(\frac{\partial r_I}{\partial t} + r_I\right) + \beta_I \left(\frac{\partial r_I}{\partial t} + r_I\right)^2 + \gamma_I \left(\frac{\partial r_I}{\partial t} + r_I\right)^3, \end{aligned} \quad \text{(S2)}$$

where

- W are the "interaction constants" reflecting the strengths of connections between the excitatory and inhibitory parts of the network ($W_{EE} = w_{EE} + 2\tilde{w}_{EE}$, $W_{EI} = w_{EI} + 2\tilde{w}_{EI}$, etc),
- and D are the "diffusion constants" reflecting the strengths of excitatory and inhibitory connections between the motifs ($D_{EE} = \tilde{w}_{EE}$, $D_{EI} = \tilde{w}_{EI}$, etc) responsible for spatial propagation of excitatory and inhibitory influences through the network.

In the equation set (S2), parameter α describes how the input current j is divided between the excitatory and inhibitory cells. That is, in a system activated by the spatiotemporal stimulus $j(x,t)$, the input currents are $i_E(x,t) = \alpha j(x,t)$ and $i_I(x,t) = (1-\alpha) j(x,t)$.

The second- and third-order Taylor expansion coefficients, β and γ, can be different for excitatory (β_E, γ_E) and inhibitory (β_I, γ_I) activation functions g_E and g_I; they define the degree of system nonlinearity. The terms that include γ control the part of response at the same frequency as the stimulus, while the terms that include β control the part responsible for higher harmonics. Since in this work we are interested in the former part of response, we keep γ terms and ignore β terms, in the interest of making results of our analysis more tractable.

Equations (S2) are key for our analysis of circuit response to spatially distributed, temporally extended stimuli.

Model of distributed computation in two spatial dimensions

In the previous section we investigated a model of one-dimensional (1D) neural chain with nearest-neighbor coupling. Such a model can describe interactions of stimuli shaped as parallel stripes or lines. To its advantage, the 1D model can be studied analytically, at least in part,

greatly simplifying the task of finding the parameters that describe qualitatively different regimes of network function.

A more general model should be able to predict responses to two-dimensional (2D) stimuli. A model of higher-dimensional circuit will allow one to investigate different network geometries and manners of coupling between motifs, offering a new and very interesting domain for analysis and simulation. Here we present an analysis of a 2D neural array, aiming to demonstrate how our approach of neural wave interference applies to such systems and how it may help interpretation of experimental results. In this analysis, we lift restrictions on neural connectivity introduced in the previous section.

For a 2D neural array, Wilson-Cowan equations can be written as:

$$\begin{aligned}\tau_E \frac{dr_E(\vec{\rho})}{dt} &= -r_E(\vec{\rho}) + g\left(\sum_{\vec{l}} \tilde{\omega}_{EE}(\vec{\rho},\vec{l}) r_E(\vec{\rho}+\vec{l}) - \sum_{\vec{l}} \tilde{\omega}_{EI}(\vec{\rho},\vec{l}) r_I(\vec{\rho}+\vec{l}) + i_E(\vec{\rho},t)\right), \\ \frac{dr_I(\vec{\rho})}{dt} &= -r_I(\vec{\rho}) + g\left(\sum_{\vec{l}} \tilde{\omega}_{IE}(\vec{\rho},\vec{l}) r_E(\vec{\rho}+\vec{l}) - \sum_{\vec{l}} \tilde{\omega}_{II}(\vec{\rho},\vec{l}) r_I(\vec{\rho}+\vec{l}) + i_I(\vec{\rho},t)\right),\end{aligned} \tag{S3}$$

where $\vec{\rho} = (x, y)$ is the location of network motif that comprises one excitatory neuron and one inhibitory neuron, and $\vec{\rho} + \vec{l} = (x + l_x, y + l_y)$ represents positions of neighboring motifs. Notice that in (S3) we sum over all motif locations, including $\vec{l} = (0, 0)$. Also, terms $\omega(\vec{\rho}, \vec{l})$ are functions representing interaction between neurons, distinct from the weights of interaction w in (S1).

To specify network geometry, we only assume that the spatial scale of neural waves is much longer than the distance between neighboring neurons. Expanding rates $r_E(\vec{\rho} + \vec{l})$ and $r_I(\vec{\rho} + \vec{l})$ in Taylor series near the point $\vec{\rho}$ allows us to derive equations describing activation of the neural tissue in the linear approximation:

$$\begin{aligned}\tau_E \frac{\partial r_E}{\partial t} &= (\mathcal{W}_{EE} - 1) r_E - \mathcal{W}_{EI} r_I + \frac{\partial}{\partial x}\left(\mathcal{W}^{(x)}_{EE} r_E - \mathcal{W}^{(x)}_{EI} r_I\right) + \frac{\partial}{\partial y}\left(\mathcal{W}^{(y)}_{EE} r_E - \mathcal{W}^{(y)}_{EI} r_I\right) \\ &+ \frac{\partial^2}{\partial x^2}\left(\mathcal{W}^{(x,x)}_{EE} r_E - \mathcal{W}^{(x,x)}_{EI} r_I\right) + \frac{\partial^2}{\partial x \partial y}\left(\mathcal{W}^{(x,y)}_{EE} r_E - \mathcal{W}^{(x,y)}_{EI} r_I\right) + \frac{\partial^2}{\partial y^2}\left(\mathcal{W}^{(y,y)}_{EE} r_E - \mathcal{W}^{(y,y)}_{EI} r_I\right) + i_E \\ \frac{\partial r_I}{\partial t} &= \mathcal{W}_{IE} r_E - (\mathcal{W}_{II} + 1) r_I + \frac{\partial}{\partial x}\left(\mathcal{W}^{(x)}_{IE} r_E - \mathcal{W}^{(x)}_{II} r_I\right) + \frac{\partial}{\partial y}\left(\mathcal{W}^{(y)}_{IE} r_E - \mathcal{W}^{(y)}_{II} r_I\right) \\ &+ \frac{\partial^2}{\partial x^2}\left(\mathcal{W}^{(x,x)}_{IE} r_E - \mathcal{W}^{(x,x)}_{II} r_I\right) + \frac{\partial^2}{\partial x \partial y}\left(\mathcal{W}^{(x,y)}_{IE} r_E - \mathcal{W}^{(x,y)}_{II} r_I\right) + \frac{\partial^2}{\partial y^2}\left(\mathcal{W}^{(y,y)}_{IE} r_E - \mathcal{W}^{(y,y)}_{II} r_I\right) + i_I\end{aligned} \tag{S4}$$

with interaction weights:

$$\mathcal{W}_{\mathcal{S}}(\vec{\rho}) = \sum_{\vec{l}} \tilde{\omega}_{\mathcal{S}}(\vec{\rho},\vec{l}); \;\; \mathcal{W}^{(x)}_{\mathcal{S}}(\vec{\rho}) = \sum_{\vec{l}} l_x \tilde{\omega}_{\mathcal{S}}(\vec{\rho},\vec{l}); \;\; \mathcal{W}^{(y)}_{\mathcal{S}}(\vec{\rho}) = \sum_{\vec{l}} l_y \tilde{\omega}_{\mathcal{S}}(\vec{\rho},\vec{l});$$

$\mathcal{W}_{\mathcal{S}}^{(x,x)}(\vec{\rho}) = \frac{1}{2}\sum_{\vec{l}} l_x^2 \tilde{\omega}_{\mathcal{S}}(\vec{\rho},\vec{l})$; $\mathcal{W}_{\mathcal{S}}^{(x,y)}(\vec{\rho}) = \sum_{\vec{l}} l_x l_y \tilde{\omega}_{\mathcal{S}}(\vec{\rho},\vec{l})$; $\mathcal{W}_{\mathcal{S}}^{(y,y)}(\vec{\rho}) = \frac{1}{2}\sum_{\vec{l}} l_y^2 \tilde{\omega}_{\mathcal{S}}(\vec{\rho},\vec{l})$,

where the subscript $\mathcal{S}$ stands for EE, EI, IE, or II.

System (S4) can represent a large class of patterns of connectivity between neurons, including multiple geometries and topologies of the network determined by the function $\tilde{\omega}_{\mathcal{S}}(\vec{l})$. The only restriction is that the waves vary on a scale that exceeds the rate of spatial decay of inter-neuronal interaction.

In general, Equations S4 describe the following features of the system:

(i) Asymmetry in propagation of of neural waves, defined by first-order derivatives $\partial/\partial x$ and $\partial/\partial y$, controlled by weights $\mathcal{W}_{\mathcal{S}}^{(x)}$ and $\mathcal{W}_{\mathcal{S}}^{(y)}$,

(ii) Anisotropic "diffusion" of activity determined by second-order derivatives $\partial^2/\partial x^2$, $\partial^2/\partial y^2$ and $\partial^2/\partial x \partial y$, controlled by weights $\mathcal{W}_{\mathcal{S}}^{(x,x)}$, $\mathcal{W}_{\mathcal{S}}^{(x,y)}$ and $\mathcal{W}_{\mathcal{S}}^{(y,y)}$.

These features are manifested in several macroscopic properties of the network:

Inhomogeneity. Inhomogeneity of neural tissue can result in dependence of $\mathcal{W}_{\mathcal{S}}$, $\mathcal{W}_{\mathcal{S}}^{(x)}$, $\mathcal{W}_{\mathcal{S}}^{(y)}$, $\mathcal{W}_{\mathcal{S}}^{(x,x)}$, $\mathcal{W}_{\mathcal{S}}^{(x,y)}$, $\mathcal{W}_{\mathcal{S}}^{(y,y)}$ on the position $\vec{\rho}$;

Asymmetry. Asymmetry can result in preferred propagation of waves in a certain direction, as in networks where response rate depends on the orientation and motion direction of the stimulus;

Diffusion anisotropy. Diffusion anisotropy can occur if, e.g., $\mathcal{W}_{\mathcal{S}}^{(2)} = \mathcal{W}_{\mathcal{S}}^{(x,x)} = \mathcal{W}_{\mathcal{S}}^{(y,y)}$ and $\mathcal{W}_{\mathcal{S}}^{(x,y)} \neq 0$, resulting in (a) elliptical traveling waves generated by short-leaved point stimuli, and (b) elliptical standing waves generated by static point stimuli, making the system sensitive to stimulus shape;

Dispersion. Inhomogeneity can result in dispersion of neural waves, which potentially allows the system to estimate the speed of signal propagation in the tissue.

In the following, our analysis is restricted to a simplified case where asymmetry, anysotropy and inhomogenities are absent, resulting in the following set of equations:

$$\begin{aligned}
\tau_E \frac{\partial r_E}{\partial t} &= (\mathcal{W}_{EE} - 1) r_E - \mathcal{W}_{EI} r_I + \left(\frac{\partial^2}{\partial x^2} + \frac{\partial^2}{\partial y^2}\right)\left(\mathcal{W}_{EE}^{(2)} r_E - \mathcal{W}_{EI}^{(2)} r_I\right) + i_E \\
\frac{\partial r_I}{\partial t} &= \mathcal{W}_{IE} r_E - (\mathcal{W}_{II} + 1) r_I + \left(\frac{\partial^2}{\partial x^2} + \frac{\partial^2}{\partial y^2}\right)\left(\mathcal{W}_{IE}^{(2)} r_E - \mathcal{W}_{II}^{(2)} r_I\right) + i_I. \quad \text{(S5)}
\end{aligned}$$

To analyze the latter set numerically, we need to select a spatial grid. Here we consider a square lattice of motifs each containing one excitatory neuron and one inhibitory neuron connected as in Figure 1a in the main text. The motifs are connected along the *sides and diagonals* of each square cells of the lattice. The relative strengths of coupling along the sides and diagonals is controlled by parameter β, which is assumed to be the same for all directions. This model can be written as

$$
\begin{aligned}
\tau_E \frac{dr_E(l,m)}{dt} &= -r_E(l,m) \\
&+ g\left(w_{EE} r_E(l,m) + \tilde{w}_{EE} \sum_E r_E - w_{EI} r_I(l,m) - \tilde{w}_{EI} \sum_I r_I + i_E(l,m,t)\right), \\
\frac{dr_I(l,m)}{dt} &= -r_I(l,m) \\
&+ g\left(w_{IE} r_E(l,m) + \tilde{w}_{IE} \sum_E r_E - w_{II} r_I(l,m) - \tilde{w}_{II} \sum_I r_I + i_I(l,m,t)\right),
\end{aligned}
\tag{S6}
$$

where

$$
\begin{aligned}
\sum_E r_E =& r_E(l+1,m) + r_E(l-1,m) + r_E(l,m+1) + r_E(l,m-1) + \\
&+ \beta[r_E(l+1,m+1) + r_E(l+1,m-1) + r_E(l-1,m+1) + r_E(l-1,m-1)]
\end{aligned}
$$

and

$$
\begin{aligned}
\sum_I r_I =& r_I(l+1,m) + r_I(l-1,m) + r_I(l,m+1) + r_I(l,m-1) + \\
&+ \beta[r_I(l+1,m+1) + r_I(l+1,m-1) + r_I(l-1,m+1) + r_I(l-1,m-1)].
\end{aligned}
$$

This model allows one to account for different classes of empirical results within a single framework. For example, an optimal set of model parameters for specific experimental results can be found by means of machine learning. An artificial neuron networks trained with the data generated by simulations with different parameter sets can constrain the model such as to best fit complementary sets of empirical observation, following an approach originally developed in the context of financial derivative modeling (Hutchinson et al., 1994).

Propagation of activity in a layered network

The general formulation of the model in Equation S4 allows one to address a range of questions concerned with propagation of neural activity in neural systems, including layered distributed networks. Here we illustrate this possibility by considering a system in which the one-dimensional neural chain shown in Figure 1B is replicated to form a

series of parallel layers. The layers are connected so that activity can propagate from one layer to another. The latter version of our model can be used to study propagation of activity from the input layer, activated by the optical stimulus, to further stages of visual information processing that include the "early" cortical areas V1 and MT.

For example, consider a static solution in the simplified case, where $\alpha = 1$ and the interaction between cells is determined by the coefficients $\mathcal{W}_{\mathcal{S}}^{(x)} = 0$, $\mathcal{W}_{EI}^{(y)} = \mathcal{W}_{IE}^{(y)} = \mathcal{W}_{II}^{(y)} = 0$, $\mathcal{W}_{EE}^{(y)} = R^{\perp}(1 + D^{\perp}(y - y_r))$ and $\mathcal{W}_{\mathcal{S}}^{(x,y)} = \mathcal{W}_{\mathcal{S}}^{(y,y)} = 0$, $\mathcal{W}_{\mathcal{S}}^{(x,x)} = D_{\mathcal{S}}$. Here, the variable x runs along neural chains (within layers), the variable y runs perpendicular to chains (between layers), and $R^{\perp}$ and $D^{\perp}$ represent the interaction between layers. In this case, activity can propagate:

(a) within layers, thanks to non-zero coupling $\mathcal{W}_{\mathcal{S}}^{(x,x)}$,

(b) between layers, thanks to non-zero interactions $\mathcal{W}_{EE}^{(y)}$.

We can therefore rewrite Equation S4 as

$$(\mathcal{W}_{EE} - 1)r_E - \mathcal{W}_{EI}r_I + \frac{\partial \mathcal{W}_{EE}^{(y)} r_E}{\partial y} + \frac{\partial^2}{\partial x^2}\left(D_{EE}r_E - D_{EI}r_I\right) + j_0 \cos(kx)\delta(y) = 0$$
$$\mathcal{W}_{IE}r_E - (\mathcal{W}_{II} + 1)r_I + \frac{\partial^2}{\partial x^2}\left(D_{IE}r_E - D_{II}r_I\right) = 0, \tag{S7}$$

where $\delta(y)$ is the Dirac function that represents direct activation of the input layer by the optical stimulus at $y = 0$. The solution has the following form:

$$r_E = \mathcal{L}_E(y)\cos(kx), \quad r_I = \mathcal{L}_I(y)\cos(kx) \tag{S8}$$

with wave amplitudes $\mathcal{L}_E(y)$ and $\mathcal{L}_I(y)$ describing the change of activation from layer to layer.

Since neurons in different layers of biological cortical networks have different properties, it is natural to assume that coefficients $D_{\mathcal{S}}(y)$ differ across layers. Natural spatial frequency is therefore expected to vary across layers, and thus resonance with stimulus frequency is expected to be attained at a certain layer

$$y = y_r.$$

We can rewrite differential equations (S7) as

$$\frac{\tilde{H}}{W_{II} + 1 - D_{II}k^2}\mathcal{L}_E = -\frac{d}{dy}\Big(R^{\perp}(1 + D^{\perp}(y - y_r))\mathcal{L}_E\Big) \approx -R^{\perp}\frac{d\mathcal{L}_E}{dy} - R^{\perp}D^{\perp}\mathcal{L}_E, \tag{S9}$$

with

$$\tilde{H} = \sqrt{\mu}\left[(k^2 - k_n^2 + \lambda^2)^2 + 4k_n^2\lambda^2\right], \tag{S10}$$

where k_n, μ, and λ are constants determined by the coefficients of interaction defined in Equations S16–S17 and S21. Near the resonance layer y_r, where $\tilde{H}$ reaches its minimum, we can approximate

$$\tilde{H} = \sqrt{\mu}[\Delta_1^2(y - y_r)^2 + \Delta_2^2],$$

where μ, Δ_2^2, and Δ_1^2 are constants determined by coefficients of interaction between cells.

With these simplifications, non-essential for qualitative analysis of the system, we can write the equation for $\mathcal{L}_E$ as

$$-\left[(y - y_r)^2 - \left(\frac{D^\perp}{\Omega} - \Delta_2^2\right)\right] \mathcal{L}_E = \frac{1}{\Omega}\frac{d\mathcal{L}_E}{dy},\ \mathcal{L}_E(y = 0) = \frac{j_0}{R^\perp} \quad \text{(S11)}$$

where Ω is a constant that depends on the coefficients of interaction near the resonance layer y_r. Under the conditions

$$y_r^2 > D^\perp/\Omega - \Delta_2^2 \ \text{ and } \ D^\perp/\Omega - \Delta_2^2 > 0,$$

the amplitude of the wave $\mathcal{L}_E$ decreases as the wave propagates between layers, and near the resonance layer, y_r, the derivative of the wave changes its sign. The amplitude then begins to increase, followed by a maximum response. An example of such a solution, with parameters

$$y_r = 1, \Omega = 15\,, \tfrac{D^\perp}{\Omega} - \Delta_2^2 = 0.3\,, \text{and } j_0 = 1,$$

is shown in Supplemental Figure 5. This example demonstrates how neural waves can propagate between network layers, while the shape of the stimulus applied to the input layer is preserved, and the amplitude of the wave is proportional to stimulus contrast. This result suggests that one can study responses of deep layers of cortical networks, removed from the retina, using the same *form* of activation as the optical stimulus.

It is also evident in Supplemental Figure 5 that the part of the layered network that is expected to be selective for stimulus frequency makes a broad band of subsequent layers. As we noted in the main text, this result is consistent with the evidence that primary visual cortex (area V1) is the largest source of input to area MT in primate visual system (Movshon & Newsome, 1996) and that neurons in areas V1 and MT are both characterized by selectivity for stimulus frequencies.

Neural wave interference within an elliptical ring

Just as in a 1D neural chain, the interference of neural waves generated in a 2D neural array may form a peculiar spatial pattern of excitation and inhibition. The pattern will consist of regions where the potential

stimuli may be facilitated or suppressed by a high-contrast inducing stimulus applied elsewhere in the 2D array.

The facilitation and suppression can be revealed by measuring the contrast threshold of a faint (low contrast) probing stimulus placed at every location in the region of interest. Indeed, if such a probe appears in the facilitated or suppressed regions, its contrast threshold should be, respectively, lower or higher than in the absence of the inducer (as in Figure 3C in the main text). This is the approach pursued by Kovács & Julesz in a seminal psychophysical study of how contrast threshold of human observers was modulated inside contours of 2D visual shapes (e.g., Figure 9C in the main text). Following the example of Kovács & Julesz, we studied the pattern of neural wave interference generated in our 2D model by a high-contrast stimulus shaped as an elliptical ring. Namely, we simulated the network response to the input $j(l,m) = 0$ for any l,m except of l,m satisfying the inequality

$$|\sqrt{l^2/R_1^2 + m^2/R_2^2} - 1| < \Delta R/\sqrt{R_1 R_2},$$

where R_1 and R_2 are semi-axes of the elliptical ring. The inputs $j(l,m)$ are represented by a dashed black ellipse in Figure 9B in the main text for (R_1, R_2) = (21,14). For the purpose of illustration, the semi-axes R_1 and R_2 were multiples of the half-period $\mathcal{L} = 7$ of neural-wave oscillation (which we measured using a point stimulus, Figure 9A), in order to obtain salient effects of constructive and destructive interference.

The pattern of neural wave interference generated by this stimulus was obtained by numerical simulation of Equations S6. The result is shown in Figure 9B in the main text. We find that, for the stimulus semi-axes $R_1 = 3\mathcal{L}$ and $R_2 = 2\mathcal{L}$, the competition between suppression and facilitation produces regions of excitation in the two foci of the elliptical ring, in agreement with a striking psychophysical result of Kovács & Julesz (1994).

Temporal dynamics

We studied response of a single motif in our model (Figure 1A in the main text) to time dependent input $j(t)$ split (as before) to excitatory and inhibitory parts: $i_E = \alpha j(t)$ and $i_I = (1-\alpha)j(t)$, where α is a fraction of the input injected in the excitatory cell.

This results in the following set of equations (*cf.* Ozeki et al., 2009):

$$\begin{aligned} \tau_E \frac{dr_E}{dt} + r_E - w_{EE} r_E + w_{EI} r_I &= \alpha j(t) \\ \frac{dr_I}{dt} + r_I - w_{IE} r_E + w_{II} r_I &= (1-\alpha) j(t). \end{aligned} \tag{S12}$$

Equation S12 just above, and Equation 1 of the main methods, are identical when inter-motif weights $\mathcal{W}$ are all set to zero.

The general solution of Equations S12 can be written by using Green's functions $G_E(t, t_0), G_I(t, t_0)$ in the following form:

$$r_E = \int_0^\infty G_E(t, t_0) j(t_0) dt_0, \quad r_I = \int_0^\infty G_I(t, t_0) j(t_0) dt_0, \tag{S13}$$

where the Green's functions satisfy the following pulse-input equations:

$$\tau_E \frac{dG_E(t, t_0)}{dt} + (1 - w_{EE}) G_E(t, t_0) + w_{EI} G_I(t, t_0) = \alpha \delta(t - t_0)$$
$$\frac{dG_I(t, t_0)}{dt} + (1 + w_{II}) G_I(t, t_0) - w_{IE} G_E(t, t_0) = (1 - \alpha) \delta(t - t_0). \tag{S14}$$

with $G_E(t < t_0, t_0) = G_I(t < t_0, t_0) = 0$. The Green functions can have the shape of a damped oscillation:

$$G_E = C e^{-\gamma(t - t_0)} \cos(\omega_f (t - t_0) - \phi), \tag{S15}$$

where

$$2\gamma = (1 - w_{EE})/\tau_E + w_{II} + 1 > 0$$

and

$$4\tau_E^2 \omega_f^2 = 4\tau_E w_{EI} w_{IE} - (\tau_E(w_{II} + 1) - 1 + w_{EE})^2 > 0.$$

Parameters γ and ω depend on weights of connections between cells, and parameters C and ϕ depend on the weight of connections and on α. Since the Green function in Equation S15 is similar to the weighting function obtained in psychophysical studies of human vision (Manahilov 1995; 1998), we used the function in Equation S15 in our analysis of duration threshold.

Using stimuli at different contrast and durations, we determined the profile of circuit response $r_E(t)$ by superposition (Equation S13) of the oscillations of r_E elicited by successive parts of the stimulus. To estimate duration threshold for a fixed input j_0 we compared the maximum of the excitatory cell response r_E with the threshold r_c, assuming that the stimulus is detected when the response exceeds r_c. Thus we obtained the equation for duration threshold, plotted in Figure 10D in the main text.

Relation between parameters responsible for the interaction of spatial and temporal stimulus frequencies

Here we consider how parameters, $k_n^2, \lambda^2, \mu, \kappa_0, \kappa_2$ and κ_0 in Equation 8 of main Methods relate to weights of interaction between cells $W_{EE}, W_{II}, W_{EI}, W_{IE}$, diffusion coefficients $D_{EE}, D_{II}, D_{EI}, D_{IE}$, and the

ratio τ_E of excitatory and inhibitory temporal constants. Following some simple but cumbersome calculations, we obtain:

$$k_n^2 = \frac{1}{2}\left\{\left(\frac{(W_{II}+1)(W_{EE}-1) - W_{EI}W_{IE}}{D_{II}D_{EE} - D_{EI}D_{IE}}\right)^{\frac{1}{2}} + \frac{(W_{II}+1)D_{EE} + (W_{EE}-1)D_{II} - D_{EI}W_{IE} - D_{IE}W_{EI}}{2(D_{II}D_{EE} - D_{EI}D_{IE})}\right\}, \tag{S16}$$

$$\lambda^2 = \frac{1}{2}\left\{\left(\frac{(W_{II}+1)(W_{EE}-1) - W_{EI}W_{IE}}{D_{II}D_{EE} - D_{EI}D_{IE}}\right)^{\frac{1}{2}} - \frac{(W_{II}+1)D_{EE} + (W_{EE}-1)D_{II} - D_{EI}W_{IE} - D_{IE}W_{EI}}{2(D_{II}D_{EE} - D_{EI}D_{IE})}\right\}, \tag{S17}$$

with the additional requirement $k_n^2 > \lambda^2 > 0$ that restricts setting of weights. We then obtain:

$$\kappa_0 = (W_{EE}-1)^2 - 2\tau_E W_{EI}W_{IE} + \tau_E^2(W_{II}+1)^2, \tag{S18}$$

$$\kappa_2 = 2(W_{EE}-1)D_{EE} - 2\tau_E^2(W_{II}+1)D_{II} - 2\tau_E(W_{EI}D_{IE} + W_{IE}D_{EI}), \tag{S19}$$

$$\kappa_4 = D_{EE}^2 - 2\tau_E D_{EI}D_{IE} + \tau_E^2 D_{II}^2, \tag{S20}$$

$$\mu = (D_{II}D_{EE} - D_{IE}D_{EI})^2. \tag{S21}$$
